# Mediterranean-Dietary Approaches to Stop Hypertension Intervention for Neurodegenerative Delay (MIND) diet and cardiovascular disease and arrhythmias

**DOI:** 10.1186/s12916-025-04546-5

**Published:** 2025-12-02

**Authors:** Pei Qin, Frederick K. Ho, Carlos A. Celis-Morales, Jill P. Pell

**Affiliations:** 1 School of Health and Wellbeing, University of Glasgow, Glasgow, UK; 2 Department of Behavioural Science and Health, University College London, London, UK; 3 School of Cardiovascular and Metabolic Health, University of Glasgow, Glasgow, UK; 4 Human Performance Lab, Education, Physical Activity and Health Research Unit, University Católica del Maule, Talca, Chile; 5 Centro de Investigación en Medicina de Altura (CEIMA), Universidad Arturo Prat, Iquique, Chile

**Keywords:** MIND diet, CVD, Arrhythmias, Cohort, Diet quality

## Abstract

**Background:**

This study aimed to explore associations between the Mediterranean-Dietary Approaches to Stop Hypertension Intervention for Neurodegenerative Delay (MIND) diet and the risk of incident cardiovascular disease (CVD) and arrhythmias, together with comparing to three other pre-existing diet quality indices.

**Methods:**

A prospective analysis was conducted using the UK Biobank. MIND diet score, Mediterranean Diet Adherence Screener (MEDAS), Recommended Food Score (RFS), and Healthy Diet Indicator (HDI) were computed using the Oxford WebQ. Cox proportional hazards regression models were used to calculate hazard ratios (HR) and 95% confidence intervals (CI).

**Results:**

193,983 participants were included in the CVD analyses, and 190,529 for arrhythmias. Compared with participants in the lowest quartile of MIND diet score, participants in the highest quartile had a lower risk of CVD (HR 0.86; 95% CI 0.81–0.91), ischemic heart disease (0.92; 0.85–0.98), stroke (0.86; 0.75–0.97), heart failure (0.79; 0.71–0.88), and total arrhythmias (0.93; 0.88–0.99), after adjusting for demographics, lifestyle, and chronic conditions. With further adjustment for metabolic profiles, the associations remain significant for CVD and its subtypes but become non-significant for arrhythmias. Strengths of association varied across diet scores, with associations for MEDAS (CVD and arrhythmias) and MIND (CVD subtypes). The associations with CVD were linear for MIND and MEDAS and non-linear for RFS. The association between MEDAS and arrhythmias was non-linear. We observed significant interactions by age and obesity for CVD.

**Conclusions:**

The MIND diet was associated with CVD and arrhythmias, relying on a single day of dietary data to derive dietary patterns. The findings suggest that following the MIND diet was associated with a lower risk of CVD, heart failure specifically, and arrhythmias.

**Supplementary Information:**

The online version contains supplementary material available at 10.1186/s12916-025-04546-5.

## Background

Diet plays a crucial role in the development of heart disease, including cardiovascular disease (CVD) and arrhythmias [1]. Research on the health effects of diet has often focused on individual foods and nutrients in isolation, which may under- or overestimate overall associations between diet and adverse health outcomes. Healthy diet scores, which combine data on nutrients and foods to describe overall dietary patterns, are increasingly used to reduce the risk of future CVD through nutrition guidelines.

The Mediterranean diet score is one of the most well-known and well-established dietary patterns in relation to cardiovascular health [2]. The Mediterranean-Dietary Approaches to Stop Hypertension Intervention for Neurodegenerative Delay (MIND) diet was developed based on healthy brain foods to focus on the neuro-protective effects of the Mediterranean diet and DASH diet [3], and has been shown to be associated with slower cognitive decline and reduced risk of dementia and Alzheimer’s disease [4], lower physical function impairment, and better muscle strength [5]. Although the MIND diet was initially derived for neurodegenerative delay, this dietary pattern has attracted recent attention as a potential strategy to improve other health outcomes [5–8]. Some recent studies evaluated other possible beneficial effects of the MIND diet on cardiovascular risk factors [6, 7] and central obesity [8]. Considering DASH and the Mediterranean diet have been reported to be associated with a lower risk of CVD [9, 10] and atrial fibrillation (AF) [11], and the MIND diet score is a combination of these two scores, it might have potential benefits on cardiovascular health and arrhythmias. However, to date, few studies have explored the association between the MIND diet and CVD [12], and research on the MIND diet and arrhythmias is lacking. Therefore, there remains a big knowledge gap regarding whether the MIND diet was associated with incident CVD and arrhythmias and their subtypes.

Moreover, there have been no studies comparing the MIND diet with other diet quality scores for CVD or arrhythmias. The findings would provide evidence to identify the beneficial dietary pattern for CVD and arrhythmia prevention. Healthy Diet Indicator (HDI) is a widely used tool to measure adherence to the World Health Organization's (WHO) nutrition guidelines [13]. RFS, which is a recent method developed to evaluate the diet quality that takes into consideration the variety of foods consumed, aligns with dietary guidelines [14]. Many large-scale observational epidemiological studies have shown associations between the HDI, the Mediterranean diet, and the Recommended Food Score (RFS), and a reduced risk of CVD [15–18].

Therefore, this study aimed to explore the associations between the MIND diet and incident CVD and arrhythmias and their subtypes, including ischemic heart disease (IHD), stroke, heart failure, AF, other arrhythmias (non-AF arrhythmias), bradyarrhythmias, and ventricular arrhythmias, and to compare these with three other diet scores (Mediterranean Diet Adherence Screener (MEDAS), RFS, HDI). We also explored the dose–response relationships between the MIND diet and other diet scores, and CVD and arrhythmias. In addition, we tested whether the associations were modified by age, sex, and obesity.

## Methods

### Study population

UK Biobank enrolled over 500,000 participants aged 37–73 years who were invited to visit one of the 22 assessment centres across England, Scotland, and Wales between 2006 and 2010 to complete touchscreen questionnaires, have physical measurements taken, and provide a biological sample [19]. All participants have provided written informed consent, and the UK Biobank study has been approved by the National Health Service and the National Research Ethics Service (Ref: 11/NW/0382). More information about the UK Biobank protocol could be found online (https://www.ukbiobank.ac.uk/.).

**Fig. 1:**
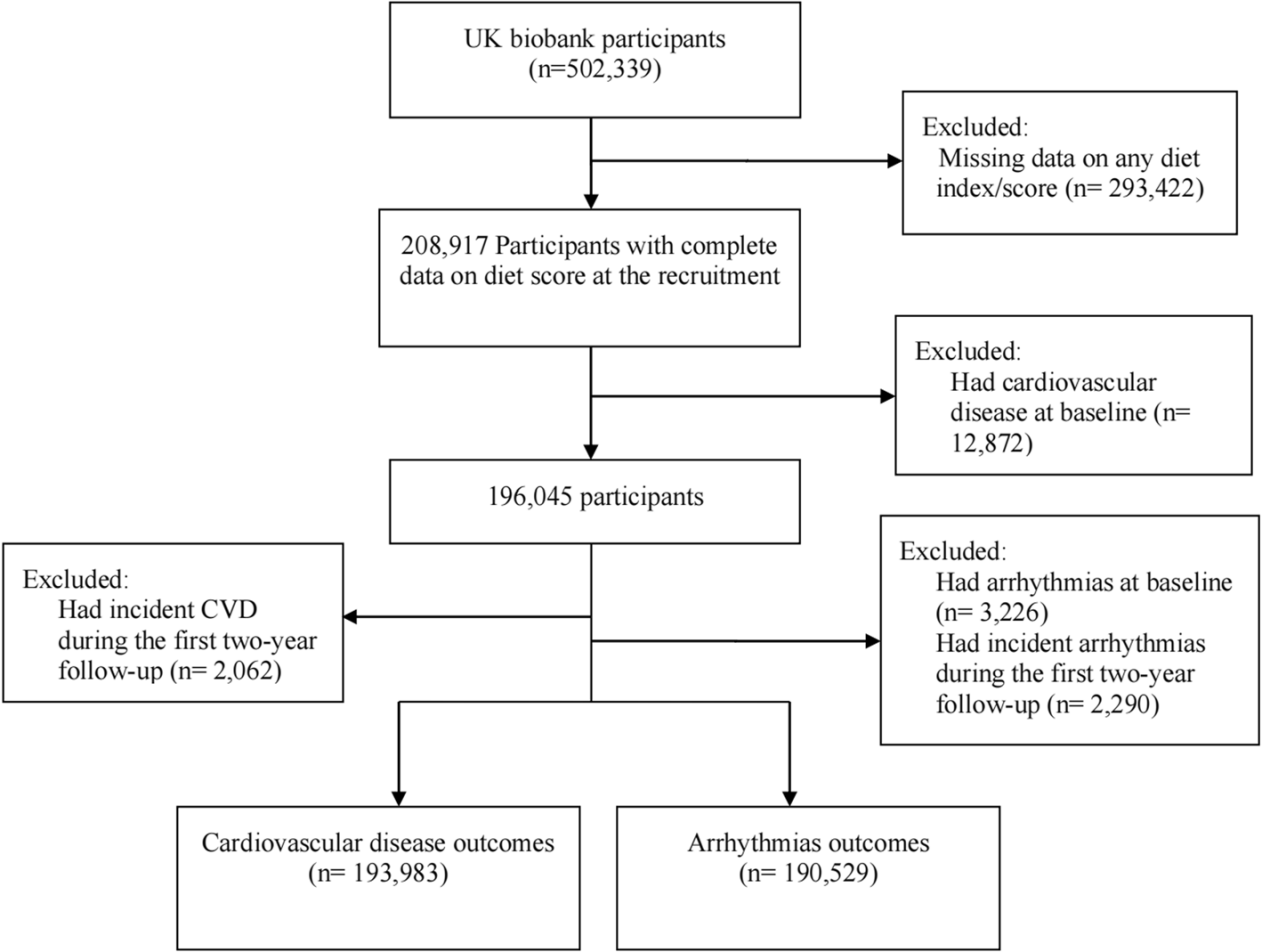
Flowchart of participant inclusion

The exclusion criteria were: (1) missing baseline data required for calculation of any diet scores (*n* = 293,422); (2) self-report of physician-diagnosed CVD (*n* = 12,731) or arrhythmia (*n* = 6052) at baseline for the CVD and arrhythmia outcomes, respectively; (3) incident CVD (*n* = 4125) or arrhythmia (*n* = 3728) in the first 2 years’ follow-up for the CVD and arrhythmia outcomes, respectively. A total of 193,983 participants were eligible for inclusion in the CVD analyses, and 190,529 for arrhythmias (Fig. 1).

### Diet scores

Dietary information was collected using the Oxford WebQ, a web-based 24-h dietary recall questionnaire [20]. The questionnaire collects information on the consumption of 206 foods and 32 beverages over the previous 24 h. UK Biobank participants were invited to complete the Oxford WebQ on five occasions over 5 years between April 2009 and June 2012, and the mean values were calculated where data were provided on more than one occasion. While a single 24-h dietary recall may not accurately reflect an individual's usual eating habits, repeated 24-h assessments can provide a more reliable estimate of habitual intake and capture potential seasonal variations in diet [21]. The Oxford WebQ has also been validated against interviewer-administered 24-h recall completed on the same day, with a Spearman rank correlation coefficient of 0.6 (range 0.5–0.9) for the majority of nutrients [22], and validated by biomarkers for protein, potassium, and total sugar intake and total energy expenditure [23]. The mean intake of main food groups using the WebQ was also shown to be consistent with the method using a touchscreen food frequency questionnaire (FFQ) [24]. We performed a sensitivity analysis by excluding those who had only completed one occasion to minimize the effects of within-person variability and random error, so that the measurement should be more representative of the actual habitual intake [25].

### Mediterranean-dietary approaches to stop hypertension intervention for neurodegenerative delay

The MIND diet score is a hybrid score of the Mediterranean and DASH diets [3] and comprises 10 brain-healthy food groups (leafy green vegetables, other vegetables, nuts, berries, legumes, whole grains, fish, poultry, olive oil, and wine) and 5 unhealthy food groups (red meat, stick butter and margarine, cheese, pastries and sweets, and fried/fast food) (see Additional file 1: Table S1 for details). For oil consumption, 1 point was allocated if olive oil was the primary oil used at home and 0 if other types of oil were used. For the other components, scores of 0, 0.5, or 1 point were assigned to each food group for meeting the whole recommended intake, half of the recommendation, or not meeting recommendations, respectively. The individual scores were summated to produce an overall diet score that ranged from 0 to 15.

### Mediterranean diet adherence screener

The MEDAS is a 14-point score used to measure adherence to the Mediterranean diet, which has previously been validated in the UK Biobank population [26]. A score of 1 was given where consumption of Mediterranean food groups (olive oil, white meats, legumes, fish, nuts, self-reported intake of tomato-based sauces, nuts, and vegetables) met or exceeded the recommended levels and where consumption of other specified food groups (commercial pastries, red meats and derivatives, carbonated beverages, and butter, margarine, or cream) was below the recommended maximum intake (Additional file 1: Table S2). The scores were summated, and the total score ranged from 0 to 14, with a higher score indicating a more Mediterranean dietary pattern.

### Recommended food score

The RFS is a food-based tally intended to evaluate the consumption of food groups that align with dietary guidelines. In the current study, the modified RFS described by Livingstone et al. in 2021 [18] was used, with a total of five food groups included in the scoring system: fruits (seven items), vegetables (seven items), whole grains (two items), meat and alternatives (three items), and reduced-fat dairy products (two items) (Additional file 1: Table S3). Participants were allocated 1 point if the consumption of food items was above the minimum thresholds (15 g/day for non-beverages and 30 g/day for beverages) and 0 points if the intake fell below these thresholds. The total score ranged from 0 to 21, with higher scores indicating better diet quality and higher consumption of recommended foods.

### Healthy diet Indicator

The HDI is a composite measure to evaluate dietary quality based on guidelines issued by the WHO, incorporating both food- and nutrient-based components. In this study, we applied a modified version of the HDI developed by Livingstone et al. [18], which uses an 11-point scoring system (Additional file 1: Table S4). This method included the following groups: saturated and polyunsaturated fats, protein, total carbohydrates, dietary fibre, fruits and vegetables, legumes and nuts, total sugars, fish, red and processed meats, and calcium. Due to the absence of detailed information on non-milk extrinsic sugars in the UK Biobank dietary data, we substituted total sugar intake in place of the original sugar component. Each component was scored as 1 if the participant’s intake met the recommended range, and 0 otherwise. The total score, ranging from 0 to 11, reflects overall adherence to healthy dietary recommendations, with higher scores representing better diet quality.

### Ascertainment of outcomes

The outcomes of interest in this study were CVD, arrhythmias, and their subtypes: IHD, stroke, and heart failure; and AF, other (i.e., non-AF) arrhythmias, bradyarrhythmias, and ventricular arrhythmias. The outcomes were ascertained from hospital admission records and death certificates, using International Statistical Classification of Diseases and Related Health Problems, Tenth Revision (ICD-10) codes (Additional file 1: Table S5).

### Covariates

The covariates included: age at baseline (calculated from date of birth and date of baseline assessment), sex (male, female), ethnicity (white, South Asian, other), Townsend deprivation index (analysed as tertiles: low, moderate, high), smoking status (never, former, current), average weekly units of alcohol consumed [27, 28], physical activity (low, moderate, high) [29], sedentary behavior (hours per day), body mass index (BMI, kg/m^2^), waist circumference (WC, cm), blood pressure (mmHg), serum total cholesterol (mmol/L), high-density lipoprotein (HDL) cholesterol (mmol/L), and number of long-term conditions (0, 1, ≥ 2) [30]. The measurement of covariates has been described in previous studies [31]. Lifestyle factors were self-reported. Total sedentary time (in hours per day) was calculated as the sum of TV viewing time, computer screen time, and leisure driving time. BMI was calculated by weight (kg)/height squared (kg/m^2^). Systolic (SBP) and diastolic (DBP) blood pressure were averaged over two repeated measurements. The total number of long-term conditions reported at baseline from a list of 47 potential conditions (other than those specified in the exclusion criteria) was categorized as 0, 1, or ≥ 2 [30]. Additional details about these measurements can be found on the UK Biobank website (https://www.ukbiobank.ac.uk.). Directed Acyclic Graph was used to explore the covariates in the present study (Additional file 1: Fig. S1).

### Statistical analyses

Baseline characteristics were summarized, broken down by outcomes using mean and standard deviation (SD) or median and interquartile range (IQR) for continuous variables, and frequency and percentage for categorical variables. Analysis of variance (ANOVA), Mann–Whitney *U*, and *χ*^2^ tests were used to test the differences between groups.

Associations between diet scores and CVD and arrhythmias were tested using Cox proportional hazard regression models, and the hazard ratios (HRs) and their 95% confidence intervals (CIs) were estimated in four models, with all diet scores being standardized and categorized into quartiles. The Schoenfeld Residuals test was used to evaluate the proportional hazard assumption, with no violation observed. Model 1 adjusted for age, sex, Townsend deprivation index, and ethnicity at baseline. Model 2 additionally adjusted for smoking status, weekly units of alcohol use, sleep duration, physical activity, total sedentary time, and total energy intake at baseline. Model 3 additionally adjusted for the number of long-term chronic diseases at baseline; Model 4 additionally adjusted for baseline HDL cholesterol level, total cholesterol level, SBP, BMI, and WC. Restricted cubic splines with three knots were used to plot the dose–response relation of the standardized diet *z* scores and CVD and arrhythmias.

We also tested for interactions with age, sex, and obesity and conducted subgroup analyses by age (< 60 and ≥ 60 years), sex (male and female), and obesity (BMI < 30 or ≥ 30) strata. Age was dichotomized at 60 years to distinguish between middle-aged and older adults, a widely used threshold in epidemiological research reflecting increased risk for age-related health outcomes [32]. Another reason to select 60 is to ensure that each subgroup has a sufficient number of participants to provide reliable results. Obesity was defined using the standard BMI cutoff of 30 kg/m^2^, based on WHO criteria [33]. A sensitivity analysis was performed, excluding participants who had only a single 24-h dietary assessment.

All analyses were conducted using R, version 4.3.2 (R Foundation for Statistical Computing) statistical packages. Two-tailed *p*-values < 0.05 were considered to indicate significance.

## Results

Over a median follow-up period of 11.2 (IQR 11.0–11.6) years, 14,259 participants developed incident CVD (9919 IHD, 2973 stroke, and 3525 heart failure), and 13,374 developed incident cardiac arrhythmias (8466 AF and 7021 other arrhythmias; 2102 bradyarrhythmias and 687 ventricular arrhythmias).

Compared to those who did not develop CVD, participants who did were more likely to be male, current smokers, less physically active, and have higher sedentary time, consume more alcohol, and have higher WC, BMI, SBP, TC levels, and more long-term conditions (Table 1). Similar between-group differences were observed for incident arrhythmias.

**Table 1:** Baseline characteristics of participants in the UK Biobank

	**Overall**	**CVD**		***P***	**Cardiac arrhythmias**		***P***
		No	Yes		No	Yes	
N	193,983	183,638	8282		175,145	13,374	
Age (years)	55.73 (7.93)	55.53 (7.92)	60.29 (6.80)	< 0.001	55.27 (7.89)	60.64 (6.53)	< 0.001
Men, *n* (%)	82,496 (43.0)	77,517 (42.2)	4979 (60.1)	< 0.001	72,886 (41.6)	7766 (58.1)	< 0.001
Deprivation index (%)				0.424			0.012
Low	68,347 (35.7)	65,452 (35.7)	2895 (35.0)		62,171 (35.5)	4897 (36.6)	
Moderate	66,096 (34.5)	63,220 (34.5)	2876 (34.8)		60,254 (34.4)	4595 (34.4)	
High	57,238 (29.9)	54,735 (29.8)	2503 (30.3)		52,495 (30.0)	3871 (29.0)	
Ethnicity (%)				0.02			< 0.001
White	183,172 (97.6)	175,205 (97.6)	7967 (98.0)		166,847 (97.5)	12,979 (98.7)	
South Asia	2050 (1.1)	1963 (1.1)	87 (1.1)		1963 (1.1)	95 (0.7)	
Other	2362 (1.3)	2287 (1.3)	75 (0.9)		2274 (1.3)	71 (0.5)	
Smoking status (%)				< 0.001			< 0.001
Never	110,300 (57.6)	106,340 (58.1)	3960 (48.0)		101,943 (58.3)	6606 (49.5)	
Previous	66,480 (34.7)	63,087 (34.4)	3393 (41.1)		59,315 (33.9)	5683 (42.6)	
Current	14,653 (7.7)	13,748 (7.5)	905 (11.0)		13,458 (7.7)	1048 (7.9)	
Alcohol, weekly units	15.76 (17.13)	15.67 (16.98)	17.79 (20.05)	< 0.001	15.53 (16.82)	18.36 (20.23)	< 0.001
Physical activity (%)				< 0.001			0.026
Low	29,347 (18.0)	27,959 (17.9)	1388 (20.1)		26,689 (17.9)	2095 (18.7)	
Moderate	69,072 (42.4)	66,317 (42.5)	2755 (40.0)		63,240 (42.5)	4625 (41.3)	
High	64,510 (39.6)	61,757 (39.6)	2753 (39.9)		58,923 (39.6)	4472 (40.0)	
Sedentary time (hours)	4.35 (2.49)	4.32 (2.48)	4.84 (2.63)	< 0.001	4.32 (2.49)	4.66 (2.53)	< 0.001
Total energy intake (kJ/day)	2123.25 (647.46)	2120.25 (645.80)	2189.85 (679.79)	< 0.001	2117.53 (645.05)	2186.34 (673.33)	< 0.001
Sleep duration (%)				< 0.001			< 0.001
1–6 h	43,056 (22.5)	41,064 (22.4)	1992 (24.2)		39,205 (22.5)	3154 (23.7)	
7–8 h	136,390 (71.3)	130,797 (71.4)	5593 (67.8)		124,840 (71.5)	9134 (68.5)	
≥ 9 h	11,874 (6.2)	11,211 (6.1)	663 (8.0)		10,560 (6.0)	1040 (7.8)	
BMI (kg/m^2^)	26.81 (4.60)	26.75 (4.57)	28.15 (5.09)	< 0.001	26.71 (4.55)	27.96 (5.04)	< 0.001
WC (cm)	88.50 (13.13)	88.24 (13.04)	94.29 (13.71)	< 0.001	88.03 (12.94)	93.68 (14.04)	< 0.001
HDL (mmol/L)	1.49 (0.38)	1.50 (0.38)	1.39 (0.37)	< 0.001	1.50 (0.38)	1.44 (0.39)	< 0.001
TG (mmol/L)	1.67 (0.98)	1.66 (0.97)	1.87 (1.05)	< 0.001	1.66 (0.98)	1.77 (1.01)	< 0.001
LDL (mmol/L)	3.60 (0.84)	3.60 (0.83)	3.60 (0.89)	0.918	3.61 (0.84)	3.55 (0.84)	< 0.001
Total cholesterol (mmol/L)	5.76 (1.09)	5.77 (1.09)	5.71 (1.17)	< 0.001	5.78 (1.09)	5.67 (1.12)	< 0.001
SBP (mmHg)	136.44 (18.24)	136.09 (18.13)	144.04 (18.99)	< 0.001	135.95 (18.11)	142.74 (18.71)	< 0.001
DBP (mmHg)	81.82 (9.98)	81.72 (9.95)	83.98 (10.36)	< 0.001	81.73 (9.96)	83.07 (10.04)	< 0.001
Number of long-term conditions (%)				< 0.001			< 0.001
0	75,555 (39.4)	73,441 (40.0)	2114 (25.5)		71,374 (40.8)	3616 (27.0)	
1	65,356 (34.1)	62,607 (34.1)	2749 (33.2)		59,664 (34.1)	4548 (34.0)	
≥ 2	51,009 (26.6)	47,590 (25.9)	3419 (41.3)		44,107 (25.2)	5210 (39.0)	
History of diabetes (%)	6472 (3.4)	5802 (3.2)	670 (8.1)	< 0.001	5448 (3.1)	887 (6.6)	< 0.001
History of hypertension (%)	41,696 (21.7)	38,635 (21.0)	3061 (37.0)	< 0.001	35,735 (20.4)	4830 (36.1)	< 0.001
History of hyperlipidemia (%)	18,115 (9.4)	16,882 (9.2)	1233 (14.9)	< 0.001	15,598 (8.9)	2017 (15.1)	< 0.001
Family history of CVD (%)	53,456 (27.9)	50,682 (27.6)	2774 (33.5)	< 0.001	47,772 (27.3)	4525 (33.8)	< 0.001
MIND score (mean (SD))	5.88 (1.07)	5.88 (1.07)	5.78 (1.06)	< 0.001	5.88 (1.07)	5.86 (1.07)	< 0.001
MEDAS score (mean (SD))	5.30 (1.98)	5.31 (1.98)	5.08 (1.96)	< 0.001	5.30 (1.98)	5.21 (1.98)	< 0.001
RFS score (mean (SD))	7.21 (2.64)	7.21 (2.64)	7.19 (2.75)	0.527	7.19 (2.64)	7.33 (2.67)	< 0.001
HDI score (mean (SD))	3.71 (1.40)	3.71 (1.40)	3.70 (1.41)	0.509	3.71 (1.40)	3.74 (1.42)	< 0.001

Abbreviations: *BMI*, body mass index; *CVD*, cardiovascular disease; *DBP*, diastolic blood pressure; *HDI*, healthy diet indicator; *HDL*, high-density lipoprotein; *LDL*, low-density lipoprotein; *MEDAS*, Mediterranean Diet Adherence; *MIND*, Mediterranean-Dietary Approaches to Stop Hypertension Intervention for Neurodegenerative Delay; *RFS*, recommended food score; *SBP*, systolic blood pressure; *TG*, triglyceride; *WC*, waist circumference

### Diet score and cardiovascular disease

**Fig. 2:**
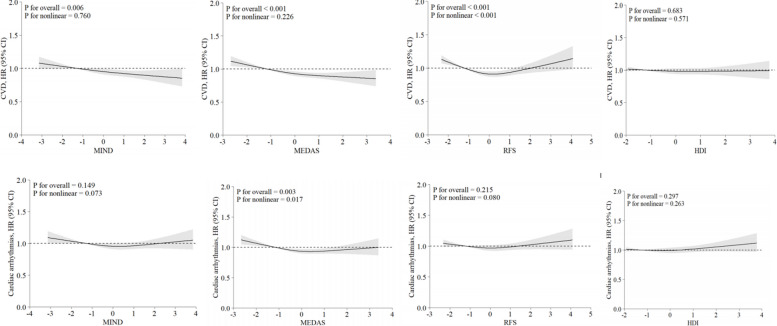
Dose–response association between standardized diet quality and incident CVD and arrhythmias. The model was adjusted for age, sex, Townsend deprivation index, ethnicity, smoking status, weekly units of alcohol use, physical activity, total sedentary time, sleep duration, total energy intake, BMI, WC, total cholesterol, SBP, number of long-term conditions at baseline. BMI, body mass index; CI, confidence interval; HDI, healthy diet indicator; HR, hazard ratio; SBP, systolic blood pressure; WC, waist circumference

After adjustment for demographic and lifestyle factors (Model 2), the highest quartiles of MIND (HR 0.85; 95% CI 0.80–0.89), MEDAS (0.80; 0.76–0.85), and RFS scores (0.83; 0.78–0.88), but not HDI score (0.98; 0.92–1.03), were significantly associated with reduced risk of incident CVD, compared to the lowest quartiles (Table 2). Further adjustment for the number of long-term conditions (Model 3) then metabolic factors (Model 4) attenuated the inverse associations, but they remained significant for all three diet scores, with the largest effect sizes observed for the MEDAS (HR: 0.87; 95% CI 0.82–0.93) and MIND scores (HR: 0.86; 95% CI 0.81–0.91) (Table 2). Significant linear dose–response relations were found for all the continuous scores except for RFS (Fig. 2). The relationships with RFS were non-linear, showing an increased risk at the lower RFS score and decreased risk in the mid-range, but no significant association at higher levels.

**Table 2:** Association of standardized diet quality and incident CVD and arrhythmias

	**Model 1**	**Model 2**	**Model 3**	**Model 4**
	**HR (95% CI)**	**HR (95% CI)**	**HR (95% CI)**	**HR (95% CI)**
**CVD**				
MIND *z* score				
Quartile 1	Ref	Ref	Ref	Ref
Quartile 2	0.92 (0.87–0.96)	0.92 (0.87–0.97)	0.93 (0.87–0.97)	0.93 (0.87–0.98)
Quartile 3	0.85 (0.81–0.90)	0.89 (0.83–0.93)	0.89 (0.84–0.94)	0.93 (0.87–0.98)
Quartile 4	0.81 (0.77–0.85)	0.85 (0.80–0.89)	0.86 (0.81–0.91)	0.90 (0.84–0.95)
MEDAS *z* score				
Quartile 1	Ref	Ref	Ref	Ref
Quartile 2	0.88 (0.84–0.92)	0.91 (0.86–0.96)	0.92 (0.86–0.96)	0.93 (0.87–0.98)
Quartile 3	0.82 (0.77–0.85)	0.86 (0.81–0.91)	0.87 (0.82–0.92)	0.91 (0.86–0.96)
Quartile 4	0.75 (0.71–0.79)	0.80 (0.76–0.85)	0.82 (0.78–0.87)	0.87 (0.82–0.93)
RFS *z* score				
Quartile 1	Ref	Ref	Ref	Ref
Quartile 2	0.82 (0.78–0.86)	0.81 (0.76–0.86)	0.87 (0.82–0.91)	0.88 (0.82–0.93)
Quartile 3	0.81 (0.77–0.85)	0.83 (0.78–0.89)	0.89 (0.84–0.94)	0.91 (0.86–0.97)
Quartile 4	0.81 (0.77–0.85)	0.83 (0.78–0.88)	0.90 (0.85–0.95)	0.93 (0.88–0.99)
HDI *z* score				
Quartile 1	Ref	Ref	Ref	Ref
Quartile 2	0.96 (0.85–1.08)	0.99 (0.93–1.04)	0.99 (0.93–1.04)	0.99 (0.93–1.04)
Quartile 3	0.94 (0.83–1.06)	0.97 (0.92–1.03)	0.98 (0.92–1.03)	1.00 (0.94–1.06)
Quartile 4	1.05 (0.93–1.18)	0.98 (0.92–1.03)	0.98 (0.92–1.03)	0.99 (0.93–1.05)
**All cardiac arrhythmias**				
MIND z score				
Quartile 1	Ref	Ref	Ref	Ref
Quartile 2	0.94 (0.89–0.99)	0.95 (0.90–1.00)	0.96 (0.90–1.01)	0.96 (0.90–1.02)
Quartile 3	0.92 (0.87–0.96)	0.94 (0.88–0.99)	0.94 (0.89–1.00)	0.98 (0.92–1.03)
Quartile 4	0.91 (0.86–0.95)	0.92 (0.87–0.97)	0.93 (0.88–0.99)	0.98 (0.92–1.04)
MEDAS *z* score				
Quartile 1	Ref	Ref	Ref	Ref
Quartile 2	0.90 (0.86–0.95)	0.91 (0.87–0.96)	0.92 (0.87–0.97)	0.94 (0.89–0.99)
Quartile 3	0.87 (0.83–0.92)	0.88 (0.84–0.93)	0.90 (0.85–0.95)	0.92 (0.87–0.98)
Quartile 4	0.85 (0.81–0.89)	0.87 (0.82–0.92)	0.89 (0.84–0.94)	0.94 (0.89–1.00)
RFS *z* score				
Quartile 1	Ref	Ref	Ref	Ref
Quartile 2	0.92 (0.88–0.97)	0.94 (0.89–0.99)	0.94 (0.89–1.00)	0.97 (0.91–1.03)
Quartile 3	0.93 (0.88–0.97)	0.94 (0.89–1.00)	0.95 (0.90–1.01)	0.99 (0.93–1.05)
Quartile 4	0.92 (0.88–0.97)	0.98 (0.92–1.03)	0.99 (0.93–1.04)	1.02 (0.96–1.09)
HDI *z* score				
Quartile 1	Ref	Ref	Ref	Ref
Quartile 2	1.03 (0.99–1.09)	1.04 (0.99–1.1)	1.04 (0.99–1.10)	1.04 (0.99–1.11)
Quartile 3	0.98 (0.94–1.03)	1.00 (0.94–1.05)	1.00 (0.95–1.06)	1.01 (0.96–1.08)
Quartile 4	0.98 (0.93–1.03)	1.02 (0.96–1.08)	1.02 (0.96–1.08)	1.03 (0.97–1.09)

Abbreviations: *Ref*, reference group; *BMI*, body mass index; *CVD*, cardiovascular disease; *CI*, confidence interval; *HDI*, healthy diet indicator; *HR*, hazard ratio; *MEDAS*, Mediterranean Diet Adherence; *MIND*, Mediterranean-Dietary Approaches to Stop Hypertension Intervention for Neurodegenerative Delay; *RFS*, recommended food score; *SBP*, systolic blood pressure; *WC*, waist circumference

Model 1: adjusted for age, sex, Townsend deprivation index, and ethnicity

Model 2: additionally adjusted for smoking status, weekly units of alcohol use, physical activity, total sedentary time, sleep duration, total energy intake

Model 3: additionally adjusted for number of long-term conditions

Model 4: additionally adjusted for BMI, WC, total cholesterol, and SBP

For the CVD subtypes, participants in the highest quartile of the MIND diet score had a lower risk of all CVD subtypes: IHD (0.92; 0.85–0.98), stroke (0.86; 0.75–0.97), and heart failure (0.79; 0.71–0.88). The other scores were less consistent. Those in the highest quartile of MEDAS had a lower risk of IHD; those in the highest quartile of RFS had a lower risk of IHD and stroke; those in the highest quartile of HDI score had a lower risk of stroke (Additional file 1: Table S7). Significant, linear dose–response relations were found for MEDAS, HDI, and IHD, and MIND and heart failure, and non-linear for RFS (Additional file 1: Fig. S2).

### Diet score and arrhythmias

In Models 1–3, compared to the lowest quartile, the highest quartile of MIND and MEDAS, but not RFS and HDI, was associated with reduced risk of total cardiac arrhythmias (Table 2). When further adjusted for potential metabolic mediators (Model 4), the associations with the highest quartiles became non-significant. Analyzing MEDAS as a continuous variable confirmed a non-linear relationship with arrhythmias (*P*_overall_ = 0.003, *P*_non-linearity_ = 0.017) (Fig. 2).

For the subtypes of arrhythmias, MIND and MEDAS scores were negatively associated with other arrhythmias, MEDAS scores were negatively associated with AF, and RFS was negatively associated with ventricular arrhythmias in models 1–3; in the full adjustment model, participants in the highest quartiles of the MIND and MEDAS scores remained to have a lower risk of other arrhythmias, but other associations became non-significant (Supplementary Table 8). There was no evidence of a non-linear relation between MEDAS and other arrhythmias (*P*_overall_ = 0.003, *P*_non-linearity_ = 0.057); there were non-linear associations of MEDAS with AF (*P*_overall_ = 0.059, *P*_non-linearity_ = 0.020) and HDI with other arrhythmias (*P*_overall_ = 0.031, *P*_non-linearity_ = 0.011) (Additional file 1: Fig. S2).

### Stratified analyses

There were statistically significant interactions between MIND score and both age (*P*_interaction_ = 0.001) and obesity (*P*_interaction_ < 0.001), whereby MIND diet was associated with a decreased risk of CVD among people aged < 60 years and those without obesity but not for those ≥ 60 years or with obesity (Table 3). Similar results were observed for both MEDAS (*P*_interaction_ = 0.004 for age and *P*_interaction_ < 0.001 for obesity) and RFS (*P*_interaction_ < 0.001 for age and obesity) with CVD (Additional file 1: Table S4). MEDAS was associated with a decreased risk of all arrhythmias among people aged < 60 years but not for those ≥ 60 years (*P*_interaction_ = 0.020). MIND was not associated with all cardiac arrhythmias in any subgroup.

**Table 3:** Association of MIND diet and incident CVD and arrhythmias

	**HR (95% CI)**				
	**Quartile 1**	**Quartile 2**	**Quartile 3**	**Quartile 4**	***P***_**interaction**_
**CVD**					
**Age**					**0.001**
< 60 years	1.00 (Ref.)	0.86 (0.79–0.94)	0.87 (0.79–0.95)	0.81 (0.73–0.89)	
≥ 60 years	1.00 (Ref.)	0.98 (0.90–1.05)	0.98 (0.91–1.05)	0.96 (0.89–1.03)	
**Sex**					0.452
Women	1.00 (Ref.)	0.90 (0.82–0.99)	0.92 (0.83–1.00)	0.85 (0.77–0.94)	
Men	1.00 (Ref.)	0.94 (0.87–1.01)	0.94 (0.87–1.01)	0.93 (0.86–1.00)	
**Obesity**					** < 0.001**
Yes	1.00 (Ref.)	0.92 (0.83–1.02)	0.95 (0.85–1.05)	0.90 (0.80–1.00)	
No	1.00 (Ref.)	0.93 (0.87–0.99)	0.92 (0.86–0.98)	0.90 (0.83–0.96)	
**All cardiac arrhythmias**					
**Age**					**0.035**
< 60 years	1.00 (Ref.)	1.02 (0.93–1.13)	0.99 (0.89–1.09)	0.95 (0.86–1.05)	
≥ 60 years	1.00 (Ref.)	0.93 (0.86–1.00)	0.98 (0.90–1.05)	1.00 (0.93–1.07)	
**Sex**					0.179
Women	1.00 (Ref.)	0.92 (0.83–1.01)	0.96 (0.87–1.06)	0.92 (0.84–1.01)	
Men	1.00 (Ref.)	0.99 (0.92–1.06)	0.99 (0.92–1.06)	1.03 (0.95–1.11)	
**Obesity**					0.790
Yes	1.00 (Ref.)	0.95 (0.85–1.06)	0.95 (0.85–1.06)	1.00 (0.89–1.12)	
No	1.00 (Ref.)	0.97 (0.90–1.04)	0.99 (0.92–1.06)	0.98 (0.91–1.05)	

Abbreviations: *Ref*, reference group; *BMI*, body mass index; *CI*, confidence interval; *CVD*, cardiovascular disease; *HR*, hazard ratio; *MIND*, Mediterranean-Dietary Approaches to Stop Hypertension Intervention for Neurodegenerative Delay; *SBP*, systolic blood pressure; *WC*, waist circumference

Model was adjusted for age, sex, Townsend deprivation index, ethnicity, smoking status, weekly units of alcohol use, physical activity, total sedentary time, sleep duration, total energy intake, number of long-term conditions, BMI, WC, total cholesterol, and SBP

### Sensitivity analysis

There were 118,235 and 116,119 participants included in the sensitivity analysis, excluding only one dietary assessment for CVD and arrhythmias associated with the diet score. In Models 1–3, compared to the lowest quartile, the higher quartiles of MIND, MEDAS, and RFS were associated with reduced risk of CVD and overall arrhythmias (Additional file 1: Table S10). When further adjusted for potential metabolic mediators (Model 4), the associations between MIND and CVD and all the significant associations for overall arrhythmias became non-significant (Additional file 1: Table S10).

## Discussion

In terms of understanding the association between diet and CVD, our findings demonstrated advantages of the MIND score over the three other diet scores: MEDAS, RFS, and HDI. Unlike the other dietary measures, high MIND scores were associated with overall CVD and all CVD sub-types (IHD, stroke, and heart failure) and all arrhythmias and other arrhythmias except for AF, independent of socioeconomic and lifestyle confounders. Metabolic factors and long-term conditions such as obesity, dyslipidaemia, and blood pressure appeared to potentially mediate the associations. The associations with CVD were specific to younger (< 60 years) and non-obese people. MEDAS was associated with the risk of all arrhythmias and arrhythmias subtypes; but for the subtypes of arrhythmias, only the MEDAS dietary scores were associated with AF, and RFS was associated with ventricular arrhythmias, independent of socioeconomic and lifestyle confounders, and both associations appeared to be mediated by obesity, dyslipidaemia, or blood pressure.

One previous cohort study, of 2,863 participants followed up for 10.6 years, reported an inverse association between the MIND diet and incident CVD [12], consistent with our findings. However, the previous study did not investigate the subtypes of CVD specifically, likely due to insufficient power since there were only CVD events in total. Furthermore, whilst the investigators adjusted for several important confounders, including energy intake and diabetes, they did not adjust for other important lifestyle factors (e.g., physical activity, alcohol intake, and sleep) that might influence dietary choices [34, 35] and are associated with increased CVD [36]. Also, they did not consider that BMI, hypertension, and lipid profile are potential mediators, rather than confounders. The present study provided new evidence by demonstrating the presence of a dose–response relationship between the MIND diet score and CVD, and that the association was specific to non-obese people and those under 60 years of age.

To our knowledge, no study has explored the relationship between the MIND diet and the overall arrhythmias and cardiac arrhythmias subtypes. Previous observational studies have reported no association between diet and AF [11, 37]. Our findings identified the significant association between the MIND diet and arrhythmias after adjusting for sociodemographic and lifestyle covariates and long-term conditions, but the association became non-significant when further adjusted for metabolic factors (MI, WC, total cholesterol, and SBP), which suggest metabolic factors as potential mediators. Using UK Biobank data, Tu et al. [11] reported that associations of AF with the DASH score and with adherence to the Mediterranean diet were explained by confounding, similar to our findings with the MIND and RFS. However, we did demonstrate a non-linear association between MEDAS and AF that was independent of sociodemographic factors, lifestyles, long-term health conditions, and metabolic factors, and appeared to be mediated through metabolic factors. The RFS score was also associated with ventricular arrhythmias specifically, which may indicate the potential benefit of the RFS score on ventricular arrhythmias.

An increasing number of diet scores have been developed, but previous studies have tended to focus on one diet score in isolation [38, 39]. Very few have compared different diet scores [12] or directly compared the strengths without standardization of the diet score [40]. In our study, we derived standardized scores of four different dietary measures, considering the different methodologies to calculate the score, and we found the varied associations of different diet scores with CVD and its subtypes. Our findings suggested that MIND may be preferable for recommendations relating to CVD and its CVD subtypes, but that MEDAS may be more appropriate for arrhythmias.

The MIND diet was originally developed for the prevention of neurodegenerative diseases [3] but is also associated with CVD [12]. This may be due to shared biological mechanisms between neurodegenerative diseases and CVD [41] and, therefore, similar protective effects of diet (e.g., consumption of berries, natural plant foods, high fiber, and a low glycemic index) on both the brain and heart. The consumption of berries, which contain abundant phenolic compounds in the MIND diet, could protect against the development of metabolic syndrome and cardiovascular complications [42]. The MIND diet considers natural plant foods and foods that are high in fiber content and have a low glycemic index, which have been shown to be inversely associated with CVD. Moreover, in contrast to other diet scores (e.g., RFS and HDI), the MIND diet contains the assessment of fast/fried foods and sweets, which are also considered to be related to CVD risk [43, 44].

The strengths of the present study included a large sample size, a prospective design with long-term follow-up, comparison of different validated and standardized diet scores, and adjustment for a wide range of potential confounders. The present study is the first comprehensive study to focus on the association of the MIND diet, in comparison with the other three diet quality scores, and CVD and arrhythmias, and provide evidence of the potential mediating role of metabolic factors and effect modifiers. The findings suggest that adherence to the MIND diet is associated with a lower risk of not only neurodegenerative decline but also heart disease.

However, a few limitations should be acknowledged. First, the diet quality scores were calculated based on self-reported 24-h diet recalls, which may be subject to recall bias and misclassification. While the Oxford WebQ is a useful tool for estimating nutrient intake in large cohorts, the dietary measurement represents only a single day's dietary intake, which does not adequately capture an individual’s habitual dietary pattern. However, short-term recalls may have less measurement error, and previous studies have validated the use of the Oxford WebQ for dietary pattern assessment, showing its comparability with FFQ, interviewer-administered 24-h recalls, and acceptable reproducibility [21–24]. This method has been widely used in previous studies to generate dietary pattern scores [45, 46]. It has been recognized that the use of single-day recall could lead to non-differential misclassification bias (also called regression dilution bias) [47]. The presence of this bias could underestimate the associations presented and therefore non-significant findings in this study do not necessarily indicate no relationship; and the estimated effect sizes are likely weaker than the true value. Moreover, weighed food records are considered the most precise quantitative dietary assessment method, requiring weighing and recording all foods and beverages consumed over a specific period (typically 3–7 days). Due to their time-consuming nature and high participant burden (e.g., literacy requirements), this method is impractical for large population cohorts like the UK Biobank. Previous studies also showed that there were no significant differences in food or nutrient intakes among the methods of weighed records and 24 h recall [48, 49] and good to strong agreement between web-based and interviewer-based 24 h dietary recall [50]. Future studies comparing weighed food records with the Oxford WebQ could thus provide valuable insights into the consistency of intake and dietary pattern estimates. Such validation would clarify the strengths and limitations of the Oxford WebQ and improve the reliability of conclusions from population-based studies using it. Second, the primary analyses in the present study included participants without missing data in any of the repeated dietary assessments, so there were participants with only one 24-h assessment; but sensitivity analyses excluding participants with only one dietary assessment yielded consistent results after adjusting for sociodemographic and lifestyle factors and multi-morbidity. Third, participants in the UK Biobank were volunteers, which may introduce healthy volunteer bias; however, previous research indicates that exposure-outcome associations are generally still applicable [51]. Since participants were aged 38–69 years and mainly of White British ethnicity, the findings may have limited generalizability to other ethnicities, regions, or age groups. Fifth, we cannot automatically infer causality from associations in observational studies, but we used a 2-year landmark analysis to minimize potential reverse causation. Sixth, the DASH score was not included for comparison with the MIND diet because it requires specific components like low-fat dairy and detailed sodium and portion size information [52], which are not reliably captured in this study's dietary assessment. Future studies comparing the MIND diet to the DASH diet are helpful in future analyses. Finally, residual confounding is a potential limitation of any observational study due to unknown or unmeasured confounders, although the present study adjusted for a wide range of confounding factors.

## Conclusions

The MIND diet was associated with CVD and subtypes (IHD, stroke, and heart failure) and arrhythmias and other arrhythmia subtypes, but not AF, relying on a single day of dietary data to derive dietary patterns. Our findings indicate that adherence to the MIND diet is associated with a lower risk of CVD, but randomized controlled trials are required to validate the findings.

## Supplementary Information

Additional file 1: Table S1 Components and scoring methods of MIND adherence score, Table S2 Components and scoring methods of MEDAS, Table S3 Components and scoring methods of RFS, Table S4 Components and scoring methods of the Healthy Diet Indicator (HDI), Table S5. ICD-10 of outcomes, Table S6 Baseline characteristics of participants in the present study sample and the entire UK Biobank, Table S7 Association of standardized diet quality and incident main CVD subtypes, Table S8 Association of standardized diet quality and incident cardiac arrhythmias subtypes, Table S9 Subgroup analyses of MEDAS, RFS, and HDI diet with incident CVD and arrhythmias, Table S10 Sensitivity analysis on the association of standardized diet quality and incident CVD and arrhythmias, Figures S1 Directed Acyclic Graph of association between diet score and CVD and arrhythmias as shown in DAGitty. Figure S2 Dose-response association between standardized diet quality and incident main CVD subtypes.

## Data Availability

The datasets used and/or analysed during the current study are available upon application to the UK Biobank from (https://www.ukbiobank.ac.uk/.).

## Abbreviations

BMI: Body mass index
CI: Confidence interval
CVD: Cardiovascular disease
DBP: Diastolic blood pressure
HDI: Healthy diet indicator
HDL: High-density lipoprotein
HDI: Healthy diet indicator
HR: Hazard ratio
LDL: Low-density lipoprotein
MEDAS: Mediterranean Diet Adherence
MIND: Mediterranean-Dietary Approaches to Stop Hypertension Intervention for Neurodegenerative Delay
RFS: Recommended food score
SBP: Systolic blood pressure
TG: Triglyceride
WC: Waist circumference
