# Supplementary material for: Mediterranean-Dietary Approaches to Stop Hypertension Intervention for Neurodegenerative Delay (MIND) diet and cardiovascular disease and arrhythmias
Source: BMC Med. 2025 Dec 2;24:13. doi: 10.1186/s12916-025-04546-5 (PMC12781738; doi:10.1186/s12916-025-04546-5)
Supplement: Supplementary file 1 — Additional file 1: Table S1 Components and scoring methods of MIND adherence score, Table S2 Components and scoring methods of MEDAS, Table S3 Components and scoring methods of RFS, Table S4 Components and scoring methods of the Healthy Diet Indicator (HDI), Table S5. ICD-10 of outcomes, Table S6 Baseline characteristics of participants in the present study sample and the entire UK Biobank, Table S7 Association of standardized diet quality and incident main CVD subtypes, Table S8 Association of standardized diet quality and incident cardiac arrhythmias subtypes, Table S9 Subgroup analyses of MEDAS, RFS, and HDI diet with incident CVD and arrhythmias, Table S10 Sensitivity analysis on the association of standardized diet quality and incident CVD and arrhythmias, Figures S1 Directed Acyclic Graph of association between diet score and CVD and arrhythmias as shown in DAGitty. Figure S2 Dose-response association between standardized diet quality and incident main CVD subtypes. [file 12916_2025_4546_MOESM1_ESM.docx]

**Additional File 1**

**Mediterranean-Dietary Approaches to Stop Hypertension Intervention for Neurodegenerative Delay (MIND) Diet and Cardiovascular Disease and Arrhythmias**

Pei Qin ^1, 2*^, Frederick K. Ho ^1^, Carlos A. Celis-Morales ^3,4,5^, Jill P. Pell ^1^

School of Health and Wellbeing, University of Glasgow, Glasgow, UK.

Department of Behavioural Science and Health, University College London, London, UK.

School of Cardiovascular and Metabolic Health, University of Glasgow, Glasgow, UK.

Human Performance Lab, Education, Physical Activity and Health Research Unit, University Católica del Maule, Talca, Chile.

Centro de Investigación en Medicina de Altura (CEIMA), Universidad Arturo Prat, Iquique, Chile.

**Corresponding Author：**

Pei Qin

Department of Behavioural Science and Health, University College London, 1-19 Torrington Place, London, WC1E 6BT, United Kingdom.

Email: p.qin@ucl.ac.uk

**Table S1.** **Components and scoring methods of MIND adherence score**

| **Diet Component** | **Contributing foods from the Oxford WebQ** | **Component Score** | | | |
| --- | --- | --- | --- | --- | --- |
|  |  | **0** | **0.5** | **1** |  |
| Green leafy vegetables | cabbage/kale, lettuce, spinach | ≤2 servings/wk | 2–6 servings/wk | ≥6 servings/wk |  |
| Other vegetables | green beans, broccoli, butternut squash, carrot, cauliﬂower, celery, cucumber, leek, mushroom, sweet pepper, sprouts, sweetcorn, sweet potato | <5 servings/wk | 5–7 servings/wk | ≥7 servings/wk |  |
| Berries | berries | <1 serving/wk | 1 serving/wk | ≥2 servings/wk |  |
| Nuts | nuts, peanuts, seeds, peanut butter | <0.5serving/wk | 0.5–4 servings/wk | >4 servings/wk |  |
| Olive oil | Participant used olive oil in cooking across all diet records that reported using fat/oil in cooking | No |  | Yes |  |
| Butter/margarine | butter/margarine on bread/crackers and potatoes | <1 tsp/d | 1–2 tsp/d | >2 tsp/d |  |
| Cheese (not low fat) | hard cheese, soft cheese, cheese spread, cottage cheese, mozzarella, goat’s cheese | ≥6 servings/wk | 1–6 servings/wk | <1 servings/wk |  |
| Whole grains | porridge, whole-wheat cereal/breads, wholemeal pasta, brown rice | <1 servings/d | 1–2 servings/d | >2 servings/d |  |
| Fish (not fried) | tinned tuna, oily ﬁsh, white ﬁsh, prawns, lobster/crab, shellﬁsh | <1 servings/m | ≥ 1 servings/m | ≥1 servings/wk |  |
| Beans | baked bean, pulses, broad bean, hummus, tofu | <1 serving/wk | 1–3 servings/wk | >3 servings/wk |  |
| Poultry (not fried) | poultry | <1 serving/wk | 1 serving/wk | ≥2servings/wk |  |
| Red meat and products | sausage, beef, pork, lamb, bacon, ham | >6 servings/wk | 4–6 servings/wk | <4 servings/wk |  |
| Fast/fried foods | fried poultry, breaded ﬁsh, fried potatoes, crisp/chips | >3 servings/wk | 1–3 servings/wk | <1 serving/wk |  |
| Pastries and sweets | pastry, crumble, pancake, pudding, ice-cream, cake, doughnut, chocolate bar, biscuits, hot chocolate, ﬁzzy drink, added sugars and preserves | ≥7 servings/wk | 5–6 servings/wk | <5 servings/wk |  |
| Wine | red wine, rose wine, white wine, fortiﬁed wine | <1 serving/m | 1 serving/m to 1 serving/wk | 2–7 servings/wk |  |

Table S2. Components and scoring methods of the MEDAS

| **Diet Component** | **Contributing foods from the Oxford WebQ** | **Component Score** | |
| --- | --- | --- | --- |
|  |  | **0** | **1** |
| Olive oil | Type of fat/ oil used for cooking | Non-consumption | Consumption |
| Vegetables | Carrot, spinach, broccoli, cabbage/ kale, sprouts, courgette, cauliflower, parsnip, turnip/ swede, leek, onion, garlic, mushroom, sweet pepper, side salad, lettuce, cucumber, celery, watercress, fresh tomato, tin tomato, sweetcorn, beetroot, avocado, mixed vegetables, vegetable pieces, butternut squash, other veg, olives, coleslaw, guacamole, vegetables from canned soup, vegetables from homemade soup | <2 servings /d | ≥2 servings /d |
| Fruit | Apple, pear, orange, satsuma, grapefruit, banana, grape, melon, peach/ nectarine, plum, berry, dried fruit, stewed fruit, mixed fruit, prune, cherry, mango, pineapple, other fruit, fruit added to cereal, grapefruit juice, orange juice | <3 servings /d | ≥3 servings /d |
| Red meat | Beef, pork, lamb, red meat from canned soup, red meat from homemade soup, bacon, ham, sausage, liver， meat from Scotch egg | ≥1 servings/d | <1 servings/d |
| Butter, margarine or cream | Butter/ margarine on potato, baguettes with butter/ margarine, baps with butter/ margarine, bread rolls with butter/ margarine, bread slices with butter/ margarine, crackers/ crispbread with butter/ margarine, oatcakes with butter/ margarine, other bread with butter/ margarine, butter/ margarine used in cooking， cream | ≥1 servings/d | <1 servings/d |
| Sweetened or carbonated drinks | Fizzy drinks, low calories drinks, squash intake | ≥1 servings/d | <1 servings/d |
| Wine | Red wine, rose wine, white wine | <7/wk | ≥7/wk |
| Legumes | Peas, green beans, broad beans, baked beans, pulses, tofu, hummus, pulses from canned soup, pulses from homemade soup | <3/wk | ≥3/wk |
| Seafood | Battered fish, breaded fish, white fish, oily fish, shellfish, other fish, tinned tuna, prawn, lobster/ crab, fish from canned soup, fish from homemade soup | <3/wk | ≥3/wk |
| Sweets or pastries | Chocolate biscuit, chocolate covered biscuit, chocolate bar, chocolate sweets, chocolate raisins, dark chocolate, milk chocolate, white chocolate, sweet biscuits, cakes, cheesecake, doughnut, fruitcake, Danish pastry, sponge pudding, milk based pudding, other milk based pudding, other desert intake, soya desert intake, sweets, diet sweets, other sweets, ice cream | ≥2/wk | <2/wk |
| Nuts | Unsalted nuts, salted nuts, unsalted peanuts, salted peanuts, peanut butter | <3/wk | ≥3/wk |
| White meat | Poultry, breaded poultry, white meat from canned soup, white meat from homemade soup | Less white meat than red meat | More white meat than red meat |
| Sofrito | Tomato-based sauce | <2/wk | ≥2/wk |

**Table S3.** **Components and scoring methods of the** **Recommended Food Score (RFS)**

| **Diet Component** | **Contributing foods from the Oxford WebQ** | **Component Score** | |
| --- | --- | --- | --- |
|  |  | **0** | **1** |
| 1. Fruits | 1. Pome fruit (apples, pears)  2. Berry fruit (berry)  3. Citrus fruit (orange, satsuma, grapefruit)  4. Stone fruit (nectarine, peach, plum, cherry, prune)  5. Tropical and subtropical fruit (banana, pineapple, mango)  6. Other fruit (other fruit, grape, melon, dried fruit, stewed fruit) | < 15 g/d | ≥15 g/d |
|  |  |  |  |
|  | 7. Fruit juice (orange juice, grapefruit juice, pure fruit/vegetable juice) | < 30 g/d | ≥30 g/d |
| 2. Vegetables | 1. Green (lettuce, spinach, sprouts, watercress, cucumber, celery, courgette) and brassica vegetables (cabbage, cauliflower, broccoli)  2. Legumes (pulses, broad bean)  3. Carrot and root vegetables (carrot, turnip/swede, beetroot parsnip, onion, garlic, leek)  4. Starchy vegetables (boiled/baked potatoes (*butter/margarine added to potatoes, butternut squash), mashed potato, sweet potato, sweetcorn)  5. Tomato and tomato products (fresh tomato, tinned tomato)  6. Peas and beans (green bean, pea)  7. Other vegetables (other vegetables, mushroom, sweet pepper, side salad, olives) | < 15 g/d | ≥15 g/d |
| 3. Whole grains | 1. Wholegrain (whole-wheat cereal, sliced bread (wholemeal), baguette (wholemeal), bap (wholemeal), bread roll (wholemeal))  2. High fibre cereals (porridge, muesli, oat crunch, bran cereal) and wholegrain pasta and brown rice | < 15 g/d | ≥15 g/d |
| 4. Lean meats and alternatives | 1. Poultry  2. Fish (tinned tuna, oily fish, white fish, prawns, lobster/crab, shellfish)  3. Alternatives (whole egg, omelette, egg in sandwich, other egg, seed (e.g. unsalted peanuts, unsalted nuts, types of spreads/sauces consumed (Peanut butter) seeds), tofu, quorn) | < 15 g/d | ≥15 g/d |
| 5. Low-fat dairy | 1. 2%, 1% or skim milk (type of milk consumed (semi skimmed, skimmed, goat/sheep milk, powdered milk, cholesterol lowering))  2. Low fat cheese and yogurt (Low fat hard cheese, low fat cheese spread, cottage cheese, yogurt (low fat yogurt consumer), goat's cheese) | < 30 g/d | ≥30 g/d |
|  |  | < 15 g/d | ≥15 g/d |

**Table S4.** **Components and scoring methods of the Healthy Diet Indicator (HDI)**

| **Diet Component** | **Contributing foods from the Oxford WebQ** | **Component Score** | |
| --- | --- | --- | --- |
|  |  | **0** | **1** |
| 1. Saturated fatty acids | Saturated fat | >10% energy intake | 0-10% energy intake |
| 2. Polyunsaturated fatty acids | Polyunsaturated fat | <6 or >10% energy intake | 6-10% energy intake |
| 3. Protein | Protein | <10 or >15% energy intake | 10-15% energy intake |
| 4. Total carbohydrates | Carbohydrates | <50% or >70% energy intake | 50-70% energy intake |
| 5. Dietary fibre | Englyst dietary fibre | <18 or >32 g/day | 18-32 g/day |
| 6. Fruits and vegetables | Mixed vegetable, vegetable pieces, avocado, beetroot, broccoli, butternut squash, cabbage/kale, carrot, cauliflower, celery, courgette, cucumber, garlic, leek, lettuce, mushroom, onion, olives, parsnip, pea, side salad, sweet pepper, spinach, sprouts, sweetcorn, fresh tomato, tinned tomato, green bean, turnip/swede, watercress, other vegetables, homemade soup (vegetables)  Stewed fruit, prune, dried fruit, mixed fruit, apple, banana, berry, cherry, grapefruit, grape, mango, melon, orange, satsuma, peach/nectarine intake, pear, pineapple, plum, other fruit | <400 g/day | ≥400 g/day |
| 7. Pulses and nuts | Baked bean, pulses, broad bean  Salted peanuts, unsalted peanuts, salted nuts, unsalted nuts, seeds, types of spreads/sauces consumed (Peanut butter) | <30 g/day | ≥30 g/day |
| 8. Total non-milk extrinsic sugars | Total sugars | >10 % energy intake | 0-10 % energy intake |
| 9. Fish | Tinned tuna, oily fish, white fish, prawns, lobster/crab, shellfish, other fish  Homemade soup, ingredients in homemade soup (fish) | <32 g/day | ≥32 g/day |
| 10. Red meat and meat products | Beef, pork, lamb, other meat  Poultry intake (skin removed from poultry (no); fat removed from poultry(no))  Homemade soup, ingredients in homemade soup (meat)  Sausage, bacon, ham, liver | >90 g/day | ≤90 g/day |
| 11. Calcium | Calcium | <700 mg/day | ≥700 mg/day |

**Table S5. ICD-10 of outcomes in this study**

| **Disease** | **ICD10** |
| --- | --- |
| CVD | I20-25, I60-I64, I50, I42.0, I42.6, I42.7, I42.9, I11.0 |
| IHD | I20-25 |
| Stroke | I60-64 |
| Heart failure | I50, I42.0, I42.6, I42.7, I42.9, I11.0 |
| Cardiac arrhythmias | I44-I49 |
| Atrial fibrillation | I48 |
| Other cardiac arrhythmias | I44-I47, I49 |
| Bradyarrhythmias | I44.0, I44.1, I44.2, I44.3, I44.5, I49.5 |
| Ventricular arrhythmias | I47.0, I47.2, I49.0, I46.0, I46.1, I46.9 |

CVD, cardiovascular disease; IHD, ischemic heart disease.

**Table S6** **Baseline characteristics of participants in the present study sample and the entire UK Biobank**

|  | **UK Biobank** | **Study sample for CVD** | **P value** | **Cohen’d/** **Cramer’s V^1^** | **Study sample for arrhythmias** | **P value** | **Cohen’d/** **Cramer’s V^1^** |
| --- | --- | --- | --- | --- | --- | --- | --- |
| N | 502339 | 193983 |  |  | 190529 |  |  |
| Age (years) | 56.53 (8.09) | 55.72 (7.94) | <0.001 | 0.101 | 55.64 (7.93) | <0.001 | 0.110 |
| Men, n (%) | 229057 (45.6) | 83780 (43.2) | <0.001 | 0.023 | 81893 (43.0) | <0.001 | 0.022 |
| Deprivation index (%) |  |  | <0.001 | 0.032 |  | <0.001 | 0.033 |
| Low | 167376 (33.4) | 69037 (35.6) |  |  | 67740 (35.6) |  |  |
| Moderate | 167150 (33.3) | 66753 (34.5) |  |  | 65491 (34.4) |  |  |
| High | 167190 (33.3) | 57949 (29.9) |  |  | 57057 (30.0) |  |  |
| Ethnicity (%) |  |  | <0.001 | 0.023 |  | <0.001 | 0.024 |
| White | 472546 (96.7) | 185056 (97.6) |  |  | 181661 (97.6) |  |  |
| South Asia | 8064 (1.7) | 2106 (1.1) |  |  | 2112 (1.1) |  |  |
| Other | 8058 (1.6) | 2424 (1.3) |  |  | 2406 (1.3) |  |  |
| Smoking status (%) |  |  | <0.001 | 0.045 |  | <0.001 | 0.045 |
| Never | 273435 (54.8) | 111555 (57.7) |  |  | 109777 (57.8) |  |  |
| Previous | 172998 (34.6) | 67062 (34.7) |  |  | 65555 (34.5) |  |  |
| Current | 52958 (10.6) | 14872 (7.7) |  |  | 14724 (7.7) |  |  |
| Alcohol, weekly units | 16.28 (18.91) | 15.65 (17.09) | <0.001 | 0.034 | 15.62 (17.05) | <0.001 | 0.035 |
| Physical activity (%) |  |  | <0.001 | 0.016 |  | <0.001 | 0.016 |
| low | 76188 (18.9) | 29743 (18.1) |  |  | 29169 (18.0) |  |  |
| moderate | 163981 (40.8) | 69744 (42.4) |  |  | 68520 (42.4) |  |  |
| high | 162084 (40.3) | 65103 (39.6) |  |  | 63971 (39.6) |  |  |
| Sedentary time (hours) | 4.51 (2.60) | 4.35 (2.50) | <0.001 | 0.062 | 4.35 (2.50) | <0.001 | 0.063 |
| Total energy intake (kJ/day) | 2116.05 (654.57) | 2121.99 (649.42) | 0.004 | -0.009 | 2121.13 (649.29) | 0.014 | -0.008 |
| Sleep duration (%) |  |  | <0.001 | 0.061 |  | <0.001 | 0.037 |
| 1-6 hours | 123205 (24.7) | 43620 (22.6) |  |  | 42905 (22.6) |  |  |
| 7-8 hours | 336582 (67.6) | 137742 (71.2) |  |  | 135294 (71.2) |  |  |
| ≥9 hours | 38338 (7.7) | 12007 (6.2) |  |  | 11730 (6.2) |  |  |
| BMI (kg/m^2^) | 27.43 (4.80) | 26.82 (4.60) | <0.001 | 0.130 | 26.81 (4.60) | <0.001 | 0.132 |
| WC (cm) | 90.31 (13.48) | 88.53 (13.13) | <0.001 | 0.133 | 88.46 (13.11) | <0.001 | 0.138 |
| HDL (mmol/L) | 1.45 (0.38) | 1.49 (0.38) | <0.001 | -0.107 | 1.49 (0.38) | <0.001 | -0.108 |
| TG (mmol/L) | 1.75 (1.03) | 1.67 (0.98) | <0.001 | 0.076 | 1.67 (0.98) | <0.001 | 0.075 |
| LDL (mmol/L) | 3.56 (0.87) | 3.60 (0.84) | <0.001 | -0.047 | 3.60 (0.84) | <0.001 | -0.053 |
| Total Cholesterol (mmol/L) | 5.69 (1.14) | 5.76 (1.10) | <0.001 | -0.062 | 5.77 (1.09) | <0.001 | -0.068 |
| SBP (mmHg) | 137.78 (18.63) | 136.42 (18.23) | <0.001 | 0.073 | 136.42 (18.23) | <0.001 | 0.073 |
| DBP (mmHg) | 82.20 (10.12) | 81.81 (9.98) | <0.001 | 0.038 | 81.82 (9.98) | <0.001 | 0.037 |
| Number of long-term conditions (%) |  |  | <0.001 | 0.067 |  | <0.001 | 0.062 |
| 0 | 173410 (34.5) | 76332 (39.3) |  |  | 75754 (39.8) |  |  |
| 1 | 164232 (32.7) | 66062 (34.1) |  |  | 64899 (34.1) |  |  |
| ≥2 | 164697 (32.8) | 51589 (26.6) |  |  | 49876 (26.2) |  |  |
| History of diabetes (%) | 25492 (5.1) | 6559 (3.4) | <0.001 | 0.036 | 6419 (3.4) | <0.001 | 0.036 |
| History of hypertension (%) | 133260 (26.5) | 42128 (21.7) | <0.001 | 0.052 | 40989 (21.5) | <0.001 | 0.050 |
| History of hyperlipidemia (%) | 61616 (12.3) | 18329 (9.4) | <0.001 | 0.041 | 17819 (9.4) | <0.001 | 0.040 |
| MIND score (mean (SD)) | 5.85 (1.08) | 5.87 (1.07) | <0.001 | -0.016 | 5.87 (1.07) | <0.001 | -0.015 |
| MEDAS score (mean (SD)) | 5.24 (1.99) | 5.28 (1.98) | <0.001 | -0.021 | 5.29 (1.98) | <0.001 | -0.021 |
| RFS score (mean (SD)) | 7.15 (2.66) | 7.18 (2.65) | <0.001 | -0.012 | 7.18 (2.65) | 0.001 | -0.011 |
| HDI score (mean (SD)) | 3.70 (1.41) | 3.70 (1.40) | 0.409 | -0.003 | 3.70 (1.40) | 0.548 | -0.002 |

Abbreviations: BMI, body mass index; CVD, cardiovascular disease; DBP, diastolic blood pressure; HDI, healthy diet indicator; HDL, high-density lipoprotein; LDL, low-density lipoprotein; MEDAS, Mediterranean Diet Adherence; MIND, Mediterranean-Dietary Approaches to Stop Hypertension Intervention for Neurodegenerative Delay; RFS, recommended food score; SBP, systolic blood pressure; TG, triglyceride; WC, waist circumference.

^1^ Effect sizes were reported as Cohen’s d for continuous variables (0.2 is small, 0.5 is medium and 0.8 is large) and Cramer’s V for categorical variables (0.1 is small, 0.3 is medium and 0.5 is large).

**Table S7 Association of standardized diet quality and incident main CVD subtypes**

|  | **Model 1** |  | **Model 2** |  | **Model 3** |  | **Model 4** |
| --- | --- | --- | --- | --- | --- | --- | --- |
|  | **HR (95% CI)** |  | **HR (95% CI)** |  | **HR (95% CI)** |  | **HR (95% CI)** |
| **IHD** |  |  |  |  |  |  |  |
| MIND z score |  |  |  |  |  |  |  |
| Quartile 1 | Ref. |  | Ref. |  | Ref. |  | Ref. |
| Quartile 2 | 0.94 (0.88-0.99) |  | 0.93 (0.87-0.99) |  | 0.94 (0.87-1.00) |  | 0.94 (0.87-1.00) |
| Quartile 3 | 0.86 (0.81-0.91) |  | 0.89 (0.83-0.95) |  | 0.90 (0.84-0.96) |  | 0.94 (0.87-1.00) |
| Quartile 4 | 0.82 (0.77-0.87) |  | 0.87 (0.81-0.93) |  | 0.88 (0.82-0.94) |  | 0.92 (0.85-0.98) |
|  |  |  |  |  |  |  |  |
| MEDAS z score |  |  |  |  |  |  |  |
| Quartile 1 | Ref. |  | Ref. |  | Ref. |  | Ref. |
| Quartile 2 | 0.88 (0.83-0.93) |  | 0.91 (0.85-0.96) |  | 0.92 (0.86-0.97) |  | 0.92 (0.86-0.98) |
| Quartile 3 | 0.82 (0.77-0.86) |  | 0.86 (0.80-0.92) |  | 0.88 (0.82-0.93) |  | 0.90 (0.84-0.96) |
| Quartile 4 | 0.73 (0.68-0.77) |  | 0.79 (0.73-0.84) |  | 0.81 (0.75-0.86) |  | 0.85 (0.79-0.91) |
|  |  |  |  |  |  |  |  |
| RFS z score |  |  |  |  |  |  |  |
| Quartile 1 | Ref. |  | Ref. |  | Ref. |  | Ref. |
| Quartile 2 | 0.81 (0.76-0.86) |  | 0.85 (0.79-0.9) |  | 0.85 (0.79-0.91) |  | 0.87 (0.81-0.93) |
| Quartile 3 | 0.79 (0.74-0.84) |  | 0.86 (0.80-0.91) |  | 0.87 (0.81-0.92) |  | 0.90 (0.84-0.97) |
| Quartile 4 | 0.79 (0.74-0.83) |  | 0.86 (0.80-0.92) |  | 0.88 (0.82-0.94) |  | 0.92 (0.85-0.98) |
|  |  |  |  |  |  |  |  |
| HDI z score |  |  |  |  |  |  |  |
| Quartile 1 | Ref. |  | Ref. |  | Ref. |  | Ref. |
| Quartile 2 | 0.93 (0.88-0.99) |  | 0.96 (0.90-1.03) |  | 0.96 (0.90-1.03) |  | 0.97 (0.90-1.04) |
| Quartile 3 | 0.95 (0.89-1.00) |  | 0.98 (0.92-1.05) |  | 0.99 (0.92-1.05) |  | 1.01 (0.94-1.08) |
| Quartile 4 | 0.93 (0.87-0.98) |  | 1.00 (0.93-1.06) |  | 1.00 (0.93-1.07) |  | 1.03 (0.95-1.10) |
|  |  |  |  |  |  |  |  |
| **Stroke** |  |  |  |  |  |  |  |
| MIND z score |  |  |  |  |  |  |  |
| Quartile 1 | Ref. |  | Ref. |  | Ref. |  | Ref. |
| Quartile 2 | 0.86 (0.77-0.95) |  | 0.87 (0.77-0.98) |  | 0.88 (0.78-0.98) |  | 0.86 (0.75-0.97) |
| Quartile 3 | 0.88 (0.79-0.97) |  | 0.89 (0.79-1.00) |  | 0.90 (0.80-1.01) |  | 0.93 (0.82-1.05) |
| Quartile 4 | 0.83 (0.75-0.92) |  | 0.83 (0.73-0.93) |  | 0.84 (0.74-0.94) |  | 0.85 (0.75-0.96) |
|  |  |  |  |  |  |  |  |
| MEDAS z score |  |  |  |  |  |  |  |
| Quartile 1 | Ref. |  | Ref. |  | Ref. |  | Ref. |
| Quartile 2 | 0.93 (0.83-1.02) |  | 0.95 (0.85-1.07) |  | 0.96 (0.85-1.07) |  | 0.98 (0.86-1.10) |
| Quartile 3 | 0.85 (0.76-0.95) |  | 0.89 (0.79-1.00) |  | 0.90 (0.80-1.02) |  | 0.94 (0.83-1.06) |
| Quartile 4 | 0.84 (0.75-0.93) |  | 0.88 (0.78-0.99) |  | 0.90 (0.79-1.01) |  | 0.94 (0.82-1.06) |
|  |  |  |  |  |  |  |  |
| RFS z score |  |  |  |  |  |  |  |
| Quartile 1 | Ref. |  | Ref. |  | Ref. |  | Ref. |
| Quartile 2 | 0.81 (0.73-0.9) |  | 0.85 (0.75-0.96) |  | 0.84 (0.74-0.95) |  | 0.84 (0.74-0.95) |
| Quartile 3 | 0.78 (0.7-0.87) |  | 0.85 (0.75-0.96) |  | 0.85 (0.75-0.97) |  | 0.85 (0.75-0.97) |
| Quartile 4 | 0.80 (0.72-0.89) |  | 0.87 (0.77-0.99) |  | 0.87 (0.76-0.99) |  | 0.88 (0.77-0.99) |
|  |  |  |  |  |  |  |  |
| HDI z score |  |  |  |  |  |  |  |
| Quartile 1 | Ref. |  | Ref. |  | Ref. |  | Ref. |
| Quartile 2 | 0.97 (0.87-1.08) |  | 1.02 (0.90-1.14) |  | 1.02 (0.90-1.14) |  | 0.97 (0.86-1.10) |
| Quartile 3 | 0.92 (0.83-1.02) |  | 0.95 (0.84-1.06) |  | 0.95 (0.84-1.07) |  | 0.96 (0.84-1.08) |
| Quartile 4 | 0.85 (0.76-0.94) |  | 0.87 (0.77-0.98) |  | 0.87 (0.77-0.99) |  | 0.82 (0.72-0.94) |
|  |  |  |  |  |  |  |  |
| **Heart failure** |  |  |  |  |  |  |  |
| MIND z score |  |  |  |  |  |  |  |
| Quartile 1 | Ref. |  | Ref. |  | Ref. |  | Ref. |
| Quartile 2 | 0.84 (0.77-0.92) |  | 0.84 (0.75-0.94) |  | 0.85 (0.76-0.94) |  | 0.87 (0.77-0.97) |
| Quartile 3 | 0.79 (0.71-0.86) |  | 0.81 (0.73-0.9) |  | 0.82 (0.73-0.91) |  | 0.87 (0.77-0.98) |
| Quartile 4 | 0.73 (0.66-0.8) |  | 0.77 (0.69-0.86) |  | 0.79 (0.71-0.88) |  | 0.87 (0.77-0.97) |
|  |  |  |  |  |  |  |  |
| MEDAS z score |  |  |  |  |  |  |  |
| Quartile 1 | Ref. |  | Ref. |  | Ref. |  | Ref. |
| Quartile 2 | 0.87 (0.79-0.95) |  | 0.88 (0.79-0.98) |  | 0.89 (0.79-0.98) |  | 0.93 (0.82-1.04) |
| Quartile 3 | 0.8 (0.72-0.87) |  | 0.86 (0.77-0.96) |  | 0.88 (0.78-0.98) |  | 0.93 (0.83-1.04) |
| Quartile 4 | 0.72 (0.65-0.79) |  | 0.79 (0.7-0.88) |  | 0.82 (0.73-0.91) |  | 0.93 (0.82-1.04) |
|  |  |  |  |  |  |  |  |
| RFS z score |  |  |  |  |  |  |  |
| Quartile 1 | Ref. |  | Ref. |  | Ref. |  | Ref. |
| Quartile 2 | 0.85 (0.77-0.93) |  | 0.93 (0.83-1.04) |  | 0.94 (0.84-1.05) |  | 0.96 (0.85-1.08) |
| Quartile 3 | 0.83 (0.75-0.92) |  | 0.91 (0.81-1.02) |  | 0.92 (0.82-1.03) |  | 0.94 (0.83-1.06) |
| Quartile 4 | 0.84 (0.76-0.92) |  | 0.97 (0.86-1.08) |  | 0.99 (0.88-1.1) |  | 1.05 (0.93-1.18) |
|  |  |  |  |  |  |  |  |
| HDI z score |  |  |  |  |  |  |  |
| Quartile 1 | Ref. |  | Ref. |  | Ref. |  | Ref. |
| Quartile 2 | 0.99 (0.90-1.09) |  | 1.03 (0.92-1.15) |  | 1.03 (0.92-1.15) |  | 1.02 (0.90-1.14) |
| Quartile 3 | 0.97 (0.88-1.07) |  | 0.99 (0.88-1.11) |  | 0.99 (0.89-1.11) |  | 1.04 (0.92-1.16) |
| Quartile 4 | 0.96 (0.87-1.05) |  | 1.01 (0.90-1.13) |  | 1.01 (0.90-1.13) |  | 1.05 (0.93-1.19) |

Abbreviations: Ref, reference group; BMI, body mass index; CI, confidence interval; CVD, cardiovascular disease; HDI, healthy diet indicator; HR, hazard ratio; IHD, ischemic heart disease; MEDAS, Mediterranean Diet Adherence; MIND, Mediterranean-Dietary Approaches to Stop Hypertension Intervention for Neurodegenerative Delay; RFS, recommended food score; SBP, systolic blood pressure; WC, waist circumference.

Model 1: adjusted for age, sex, Townsend deprivation index, and ethnicity;

Model 2: additionally adjusted for smoking status, weekly units of alcohol use, physical activity, total sedentary time, sleep duration, total energy intake;

Model 3: additionally adjusted for number of long-term conditions.

Model 4: additionally adjusted for BMI, WC, total cholesterol, and SBP.

**Table S8 Association of standardized diet quality and incident cardiac arrhythmias subtypes**

|  | **Model 1** | **Model 2** | **Model 3** | **Model 4** |
| --- | --- | --- | --- | --- |
|  | **HR (95% CI)** | **HR (95% CI)** | **HR (95% CI)** | **HR (95% CI)** |
| **Atrial Fibrillation** |  |  |  |  |
| MIND z score |  |  |  |  |
| Quartile 1 | Ref. | Ref. | Ref. | Ref. |
| Quartile 2 | 0.94 (0.88-0.99) | 0.95 (0.89-1.01) | 0.96 (0.90-1.02) | 0.98 (0.91-1.05) |
| Quartile 3 | 0.94 (0.89-1.00) | 0.94 (0.89-1.01) | 0.95 (0.90-1.02) | 1.02 (0.95-1.10) |
| Quartile 4 | 0.94 (0.89-1.00) | 0.94 (0.89-1.00) | 0.96 (0.90-1.02) | 1.05 (0.98-1.13) |
|  |  |  |  |  |
| MEDAS z score |  |  |  |  |
| Quartile 1 | Ref. | Ref. | Ref. | Ref. |
| Quartile 2 | 0.90 (0.84-0.95) | 0.90 (0.85-0.97) | 0.91 (0.85-0.97) | 0.93 (0.87-1.00) |
| Quartile 3 | 0.88 (0.83-0.94) | 0.89 (0.83-0.96) | 0.90 (0.84-0.97) | 0.95 (0.88-1.02) |
| Quartile 4 | 0.88 (0.82-0.93) | 0.89 (0.84-0.96) | 0.91 (0.85-0.98) | 0.99 (0.92-1.07) |
|  |  |  |  |  |
| RFS z score |  |  |  |  |
| Quartile 1 | Ref. | Ref. | Ref. | Ref. |
| Quartile 2 | 0.91 (0.85-0.96) | 0.93 (0.87-1.00) | 0.94 (0.87-1.00) | 0.97 (0.90-1.04) |
| Quartile 3 | 0.95 (0.89-1.01) | 0.99 (0.92-1.06) | 0.99 (0.93-1.07) | 1.04 (0.97-1.12) |
| Quartile 4 | 0.94 (0.88-0.99) | 0.99 (0.92-1.07) | 1.00 (0.93-1.07) | 1.06 (0.98-1.14) |
|  |  |  |  |  |
| HDI z score |  |  |  |  |
| Quartile 1 | Ref. | Ref. | Ref. | Ref. |
| Quartile 2 | 1.10 (1.04-1.17) | 1.12 (1.05-1.2) | 1.12 (1.05-1.2) | 1.14 (1.06-1.23) |
| Quartile 3 | 0.98 (0.92-1.04) | 1.01 (0.94-1.08) | 1.01 (0.94-1.08) | 1.04 (0.96-1.12) |
| Quartile 4 | 1.01 (0.95-1.07) | 1.06 (0.98-1.13) | 1.06 (0.99-1.14) | 1.08 (1.01-1.17) |
|  |  |  |  |  |
| **Other arrhythmias** |  |  |  |  |
| MIND z score |  |  |  |  |
| Quartile 1 | Ref. | Ref. | Ref. | Ref. |
| Quartile 2 | 0.95 (0.89-1.02) | 0.96 (0.89-1.03) | 0.96 (0.89-1.04) | 0.97 (0.90-1.05) |
| Quartile 3 | 0.90 (0.84-0.96) | 0.92 (0.86-0.99) | 0.93 (0.86-1.00) | 0.96 (0.89-1.04) |
| Quartile 4 | 0.87 (0.81-0.93) | 0.88 (0.82-0.95) | 0.90 (0.83-0.97) | 0.92 (0.85-0.99) |
|  |  |  |  |  |
| MEDAS z score |  |  |  |  |
| Quartile 1 | Ref. | Ref. | Ref. | Ref. |
| Quartile 2 | 0.90 (0.85-0.96) | 0.93 (0.86-1.00) | 0.94 (0.87-1.01) | 0.94 (0.87-1.01) |
| Quartile 3 | 0.87 (0.81-0.93) | 0.89 (0.83-0.96) | 0.91 (0.84-0.98) | 0.91 (0.84-0.98) |
| Quartile 4 | 0.83 (0.78-0.89) | 0.86 (0.80-0.93) | 0.89 (0.82-0.96) | 0.92 (0.85-0.99) |
|  |  |  |  |  |
| RFS z score |  |  |  |  |
| Quartile 1 | Ref. | Ref. | Ref. | Ref. |
| Quartile 2 | 0.93 (0.87-1.00) | 0.95 (0.88-1.03) | 0.96 (0.88-1.03) | 0.97 (0.90-1.05) |
| Quartile 3 | 0.91 (0.85-0.98) | 0.93 (0.86-1.01) | 0.94 (0.87-1.02) | 0.97 (0.89-1.05) |
| Quartile 4 | 0.92 (0.86-0.99) | 0.99 (0.91-1.07) | 1.00 (0.92-1.08) | 1.02 (0.94-1.11) |
|  |  |  |  |  |
| HDI z score |  |  |  |  |
| Quartile 1 | Ref. | Ref. | Ref. | Ref. |
| Quartile 2 | 1.00 (0.93-1.07) | 1.02 (0.94-1.10) | 1.02 (0.94-1.1) | 0.99 (0.91-1.08) |
| Quartile 3 | 0.98 (0.92-1.05) | 0.99 (0.92-1.07) | 0.99 (0.92-1.07) | 0.99 (0.92-1.08) |
| Quartile 4 | 0.96 (0.89-1.03) | 0.99 (0.91-1.07) | 0.99 (0.91-1.07) | 0.98 (0.90-1.07) |
|  |  |  |  |  |
| **Bradyarrhythmias** |  |  |  |  |
| MIND z score |  |  |  |  |
| Quartile 1 | Ref. | Ref. | Ref. | Ref. |
| Quartile 2 | 0.96 (0.79-1.17) | 0.96 (0.76-1.21) | 0.96 (0.76-1.21) | 1.06 (0.83-1.35) |
| Quartile 3 | 0.72 (0.58-0.89) | 0.73 (0.57-0.94) | 0.73 (0.57-0.94) | 0.81 (0.62-1.05) |
| Quartile 4 | 0.71 (0.57-0.89) | 0.80 (0.63-1.02) | 0.81 (0.64-1.04) | 0.86 (0.66-1.12) |
|  |  |  |  |  |
| MEDAS z score |  |  |  |  |
| Quartile 1 | Ref. | Ref. | Ref. | Ref. |
| Quartile 2 | 0.88 (0.78-0.99) | 0.89 (0.77-1.02) | 0.92 (0.80-1.06) | 0.92 (0.80-1.06) |
| Quartile 3 | 0.92 (0.81-1.03) | 0.95 (0.83-1.09) | 0.98 (0.85-1.13) | 0.98 (0.85-1.13) |
| Quartile 4 | 0.89 (0.79-1.00) | 0.94 (0.82-1.08) | 1.01 (0.88-1.17) | 1.01 (0.88-1.17) |
|  |  |  |  |  |
| RFS z score |  |  |  |  |
| Quartile 1 | Ref. | Ref. | Ref. | Ref. |
| Quartile 2 | 0.88 (0.77-0.99) | 0.86 (0.75-0.99) | 0.86 (0.75-1.00) | 0.88 (0.76-1.03) |
| Quartile 3 | 0.92 (0.81-1.04) | 0.95 (0.82-1.09) | 0.95 (0.83-1.10) | 1.01 (0.87-1.17) |
| Quartile 4 | 0.98 (0.87-1.11) | 1.04 (0.90-1.19) | 1.05 (0.91-1.21) | 1.08 (0.93-1.26) |
|  |  |  |  |  |
| HDI z score |  |  |  |  |
| Quartile 1 | Ref. | Ref. | Ref. | Ref. |
| Quartile 2 | 0.99 (0.87-1.12) | 1.05 (0.91-1.21) | 1.05 (0.91-1.21) | 1.07 (0.92-1.24) |
| Quartile 3 | 1.00 (0.89-1.13) | 1.04 (0.90-1.2) | 1.04 (0.91-1.20) | 1.08 (0.94-1.26) |
| Quartile 4 | 1.00 (0.88-1.12) | 1.04 (0.89-1.19) | 1.04 (0.90-1.20) | 1.05 (0.90-1.22) |
|  |  |  |  |  |
| **Ventricular arrhythmias** |  |  |  |  |
| MIND z score |  |  |  |  |
| Quartile 1 | Ref. | Ref. | Ref. | Ref. |
| Quartile 2 | 1.00 (0.89-1.13) | 0.98 (0.86-1.12) | 0.98 (0.86-1.13) | 0.97 (0.84-1.12) |
| Quartile 3 | 0.94 (0.84-1.06) | 0.93 (0.81-1.06) | 0.93 (0.81-1.07) | 0.96 (0.83-1.11) |
| Quartile 4 | 0.91 (0.81-1.03) | 0.89 (0.78-1.02) | 0.90 (0.79-1.04) | 0.92 (0.79-1.06) |
|  |  |  |  |  |
| MEDAS z score |  |  |  |  |
| Quartile 1 | Ref. | Ref. | Ref. | Ref. |
| Quartile 2 | 0.93 (0.76-1.13) | 0.92 (0.73-1.16) | 0.92 (0.73-1.16) | 0.95 (0.74-1.22) |
| Quartile 3 | 0.75 (0.61-0.93) | 0.84 (0.66-1.07) | 0.85 (0.67-1.08) | 0.88 (0.68-1.14) |
| Quartile 4 | 0.68 (0.55-0.85) | 0.79 (0.62-1.02) | 0.82 (0.64-1.05) | 0.90 (0.69-1.17) |
|  |  |  |  |  |
| RFS z score |  |  |  |  |
| Quartile 1 | Ref. | Ref. | Ref. | Ref. |
| Quartile 2 | 0.79 (0.64-0.97) | 0.86 (0.67-1.09) | 0.85 (0.67-1.09) | 0.92 (0.72-1.19) |
| Quartile 3 | 0.76 (0.62-0.94) | 0.88 (0.69-1.12) | 0.88 (0.69-1.12) | 0.94 (0.73-1.22) |
| Quartile 4 | 0.64 (0.52-0.80) | 0.77 (0.59-0.99) | 0.77 (0.60-1.00) | 0.73 (0.56-0.97) |
|  |  |  |  |  |
| HDI z score |  |  |  |  |
| Quartile 1 | Ref. | Ref. | Ref. | Ref. |
| Quartile 2 | 1.00 (0.81-1.24) | 1.13 (0.87-1.45) | 1.14 (0.88-1.46) | 1.10 (0.84-1.44) |
| Quartile 3 | 0.97 (0.78-1.20) | 1.12 (0.87-1.45) | 1.12 (0.87-1.45) | 1.13 (0.86-1.48) |
| Quartile 4 | 0.95 (0.76-1.18) | 1.17 (0.90-1.50) | 1.17 (0.90-1.51) | 1.17 (0.88-1.53) |

Abbreviations: Ref, reference group; BMI, body mass index; CI, confidence interval; HDI, healthy diet indicator; HR, hazard ratio; CVD, cardiovascular disease; MEDAS, Mediterranean Diet Adherence; MIND, Mediterranean-Dietary Approaches to Stop Hypertension Intervention for Neurodegenerative Delay; RFS, recommended food score; SBP, systolic blood pressure; WC, waist circumference.

Model 1: adjusted for age, sex, Townsend deprivation index, and ethnicity;

Model 2: additionally adjusted for smoking status, weekly units of alcohol use, physical activity, total sedentary time, sleep duration, total energy intake;

Model 3: additionally adjusted for number of long-term conditions.

Model 4: additionally adjusted for BMI, WC, total cholesterol, and SBP.

**Table S9 Subgroup analyses of MEDAS, RFS, and HDI diet with incident CVD and arrhythmias**

|  | **Quartile 1** | **Quartile 2** | **Quartile 3** | **Quartile 4** | **P interaction** |
| --- | --- | --- | --- | --- | --- |
| **MEDAS** |  |  |  |  |  |
| **CVD** |  |  |  |  |  |
| **Age** |  |  |  |  | **0.004** |
| **<**60 years | 1.00 (Ref.) | 0.88 (0.81-0.96) | 0.83 (0.75-0.90) | 0.80 (0.73-0.88) |  |
| ≥ 60 years | 1.00 (Ref.) | 0.97 (0.90-1.04) | 0.98 (0.91-1.05) | 0.93 (0.86-1.00) |  |
| **Sex** |  |  |  |  | 0.968 |
| Women | 1.00 (Ref.) | 0.93 (0.84-1.02) | 0.90 (0.82-0.99) | 0.88 (0.79-0.97) |  |
| Men | 1.00 (Ref.) | 0.93 (0.87-1.00) | 0.92 (0.85-0.98) | 0.88 (0.81-0.94) |  |
| **Obesity** |  |  |  |  | **<0.001** |
| Yes | 1.00 (Ref.) | 0.89 (0.81-0.99) | 0.91 (0.81-1.01) | 0.87 (0.77-0.97) |  |
| No | 1.00 (Ref.) | 0.95 (0.88-1.01) | 0.92 (0.85-0.98) | 0.88 (0.82-0.94) |  |
|  |  |  |  |  |  |
| **All cardiac arrhythmias** |  |  |  |  |  |
| **Age** |  |  |  |  | **0.020** |
| **<**60 years | 1.00 (Ref.) | 0.95 (0.86-1.04) | 0.86 (0.78-0.95) | 0.87 (0.79-0.97) |  |
| ≥ 60 years | 1.00 (Ref.) | 0.94 (0.87-1.01) | 0.96 (0.89-1.03) | 0.98 (0.91-1.06) |  |
| **Sex** |  |  |  |  | 0.308 |
| Women | 1.00 (Ref.) | 0.91 (0.82-1.00) | 0.89 (0.81-0.98) | 0.89 (0.80-0.98) |  |
| Men | 1.00 (Ref.) | 0.95 (0.88-1.02) | 0.94 (0.87-1.01) | 0.98 (0.91-1.06) |  |
| **Obesity** |  |  |  |  | 0.752 |
| Yes | 1.00 (Ref.) | 0.92 (0.82-1.02) | 0.95 (0.85-1.05) | 0.93 (0.82-1.04) |  |
| No | 1.00 (Ref.) | 0.94 (0.88-1.01) | 0.91 (0.85-0.98) | 0.95 (0.88-1.01) |  |
|  |  |  |  |  |  |
| **RFS** |  |  |  |  |  |
| **CVD** |  |  |  |  |  |
| **Age** |  |  |  |  | **<0.001** |
| **<**60 years | 1.00 (Ref.) | 0.81 (0.74-0.88) | 0.84 (0.77-0.92) | 0.85 (0.77-0.93) |  |
| ≥ 60 years | 1.00 (Ref.) | 0.95 (0.87-1.02) | 0.98 (0.91-1.06) | 1.00 (0.93-1.08) |  |
| **Sex** |  |  |  |  | 0.763 |
| Women | 1.00 (Ref.) | 0.86 (0.77-0.96) | 0.93 (0.83-1.02) | 0.91 (0.82-1.01) |  |
| Men | 1.00 (Ref.) | 0.89 (0.83-0.95) | 0.91 (0.84-0.97) | 0.95 (0.88-1.02) |  |
| **Obesity** |  |  |  |  | **<0.001** |
| Yes | 1.00 (Ref.) | 0.86 (0.77-0.95) | 0.90 (0.80-1) | 0.97 (0.86-1.08) |  |
| No | 1.00 (Ref.) | 0.88 (0.82-0.95) | 0.92 (0.86-0.98) | 0.93 (0.86-0.99) |  |
|  |  |  |  |  |  |
| **All cardiac arrhythmias** |  |  |  |  |  |
| **Age** |  |  |  |  | 0.134 |
| **<**60 years | 1.00 (Ref.) | 0.91 (0.83-1.00) | 0.95 (0.86-1.05) | 0.99 (0.89-1.10) |  |
| ≥ 60 years | 1.00 (Ref.) | 1.01 (0.94-1.10) | 1.03 (0.95-1.11) | 1.05 (0.97-1.14) |  |
| **Sex** |  |  |  |  | 0.213 |
| Women | 1.00 (Ref.) | 0.96 (0.86-1.07) | 0.95 (0.86-1.05) | 0.96 (0.86-1.06) |  |
| Men | 1.00 (Ref.) | 0.97 (0.90-1.04) | 1.01 (0.93-1.09) | 1.07 (0.99-1.16) |  |
| **Obesity** |  |  |  |  | 0.504 |
| Yes | 1.00 (Ref.) | 0.98 (0.88-1.09) | 1.02 (0.91-1.14) | 0.99 (0.88-1.11) |  |
| No | 1.00 (Ref.) | 0.96 (0.89-1.03) | 0.98 (0.91-1.05) | 1.03 (0.95-1.10) |  |
|  |  |  |  |  |  |
| **HDI** |  |  |  |  |  |
| **CVD** |  |  |  |  |  |
| **Age** |  |  |  |  | 0.203 |
| **<**60 years | 1.00 (Ref.) | 0.99 (0.90-1.07) | 0.97 (0.88-1.06) | 0.97 (0.88-1.07) |  |
| ≥ 60 years | 1.00 (Ref.) | 0.99 (0.91-1.06) | 1.02 (0.94-1.1) | 1.01 (0.93-1.08) |  |
| **Sex** |  |  |  |  | 0.167 |
| Women | 1.00 (Ref.) | 1.01 (0.92-1.12) | 1.03 (0.93-1.13) | 1.06 (0.96-1.16) |  |
| Men | 1.00 (Ref.) | 0.97 (0.90-1.04) | 0.99 (0.91-1.06) | 0.95 (0.88-1.03) |  |
| **Obesity** |  |  |  |  | **<0.001** |
| Yes | 1.00 (Ref.) | 0.91 (0.82-1.01) | 0.91 (0.82-1.02) | 0.96 (0.85-1.07) |  |
| No | 1.00 (Ref.) | 1.02 (0.95-1.09) | 1.04 (0.96-1.11) | 1.01 (0.94-1.09) |  |
| **All cardiac arrhythmias** |  |  |  |  |  |
| **Age** |  |  |  |  | 0.256 |
| **<**60 years | 1.00 (Ref.) | 1.10 (1.00-1.21) | 1.00 (0.90-1.11) | 1.10 (0.99-1.22) |  |
| ≥ 60 years | 1.00 (Ref.) | 1.01 (0.94-1.09) | 1.02 (0.94-1.09) | 0.99 (0.92-1.07) |  |
| **Sex** |  |  |  |  | 0.038 |
| Women | 1.00 (Ref.) | 0.95 (0.86-1.05) | 0.92 (0.83-1.01) | 0.94 (0.85-1.03) |  |
| Men | 1.00 (Ref.) | 1.10 (1.02-1.18) | 1.07 (0.99-1.16) | 1.08 (1.00-1.17) |  |
| **Obesity** |  |  |  |  | 0.102 |
| Yes | 1.00 (Ref.) | 0.96 (0.85-1.06) | 0.91 (0.81-1.02) | 0.99 (0.88-1.11) |  |
| No | 1.00 (Ref.) | 1.08 (1.01-1.16) | 1.05 (0.98-1.13) | 1.04 (0.97-1.12) |  |

The model was adjusted for age, sex, Townsend deprivation index, ethnicity, smoking status, weekly units of alcohol use, physical activity, total sedentary time, sleep duration, total energy intake, BMI, WC, total cholesterol, SBP, number of long-term conditions at baseline. BMI, body mass index; CI, confidence interval; HDI, healthy diet indicator; HR, hazard ratio; MEDAS, Mediterranean Diet Adherence; RFS, recommended food score; SBP, systolic blood pressure; WC, waist circumference.

**Table S10 Sensitivity analysis on the association of standardized diet quality and incident CVD and arrythmias**

|  | **Model 1** | **Model 2** | **Model 3** | **Model 4** |
| --- | --- | --- | --- | --- |
|  | **HR (95% CI)** | **HR (95% CI)** | **HR (95% CI)** | **HR (95% CI)** |
| **CVD** |  |  |  |  |
| MIND z score |  |  |  |  |
| Quartile 1 | Ref. | Ref. | Ref. | Ref. |
| Quartile 2 | 0.94 (0.88-0.99) | 0.96 (0.89-1.03) | 0.96 (0.89-1.03) | 0.98 (0.91-1.06) |
| Quartile 3 | 0.88 (0.82-0.94) | 0.91 (0.84-0.97) | 0.91 (0.84-0.98) | 0.96 (0.89-1.03) |
| Quartile 4 | 0.84 (0.78-0.89) | 0.88 (0.81-0.94) | 0.89 (0.83-0.96) | 0.94 (0.86-1.01) |
|  |  |  |  |  |
| MEDAS z score |  |  |  |  |
| Quartile 1 | Ref. | Ref. | Ref. | Ref. |
| Quartile 2 | 0.86 (0.81-0.91) | 0.88 (0.81-0.94) | 0.89 (0.82-0.95) | 0.91 (0.84-0.97) |
| Quartile 3 | 0.82 (0.77-0.88) | 0.86 (0.8-0.93) | 0.88 (0.82-0.95) | 0.93 (0.86-1.00) |
| Quartile 4 | 0.75 (0.70-0.80) | 0.8 (0.74-0.85) | 0.82 (0.76-0.88) | 0.88 (0.81-0.94) |
|  |  |  |  |  |
| RFS z score |  |  |  |  |
| Quartile 1 | Ref. | Ref. | Ref. | Ref. |
| Quartile 2 | 0.82 (0.77-0.87) | 0.87 (0.80-0.93) | 0.87 (0.81-0.94) | 0.89 (0.82-0.96) |
| Quartile 3 | 0.85 (0.79-0.90) | 0.91 (0.84-0.98) | 0.92 (0.85-0.99) | 0.94 (0.87-1.02) |
| Quartile 4 | 0.81 (0.76-0.86) | 0.88 (0.81-0.94) | 0.89 (0.82-0.96) | 0.93 (0.86-1.00) |
|  |  |  |  |  |
| HDI z score |  |  |  |  |
| Quartile 1 | Ref. | Ref. | Ref. | Ref. |
| Quartile 2 | 0.94 (0.88-1.00) | 0.98 (0.91-1.06) | 0.98 (0.91-1.05) | 0.98 (0.90-1.05) |
| Quartile 3 | 0.93 (0.87-0.98) | 0.96 (0.89-1.03) | 0.96 (0.89-1.03) | 0.99 (0.91-1.07) |
| Quartile 4 | 0.90 (0.84-0.96) | 0.95 (0.88-1.03) | 0.96 (0.89-1.03) | 0.99 (0.91-1.07) |
|  |  |  |  |  |
| **Arrhythmias** |  |  |  |  |
| MIND z score |  |  |  |  |
| Quartile 1 | Ref. | Ref. | Ref. | Ref. |
| Quartile 2 | 0.93 (0.87-0.99) | 0.94 (0.87-1.01) | 0.94 (0.87-1.01) | 0.96 (0.88-1.03) |
| Quartile 3 | 0.93 (0.87-0.99) | 0.93 (0.86-0.99) | 0.93 (0.86-1.00) | 0.98 (0.90-1.06) |
| Quartile 4 | 0.94 (0.88-1.00) | 0.94 (0.87-1.01) | 0.95 (0.88-1.02) | 1.00 (0.92-1.08) |
|  |  |  |  |  |
| MEDAS z score |  |  |  |  |
| Quartile 1 | Ref. | Ref. | Ref. | Ref. |
| Quartile 2 | 0.92 (0.86-0.97) | 0.93 (0.86-0.99) | 0.93 (0.87-1.00) | 0.96 (0.89-1.03) |
| Quartile 3 | 0.88 (0.82-0.94) | 0.89 (0.82-0.95) | 0.90 (0.83-0.96) | 0.93 (0.85-1.00) |
| Quartile 4 | 0.90 (0.84-0.96) | 0.91 (0.84-0.98) | 0.93 (0.86-1.00) | 0.99 (0.92-1.07) |
|  |  |  |  |  |
| RFS z score |  |  |  |  |
| Quartile 1 | Ref. | Ref. | Ref. | Ref. |
| Quartile 2 | 0.94 (0.87-1.00) | 0.93 (0.86-1.00) | 0.93 (0.86-1.01) | 0.94 (0.86-1.01) |
| Quartile 3 | 0.95 (0.89-1.02) | 0.94 (0.87-1.01) | 0.95 (0.87-1.02) | 0.98 (0.90-1.06) |
| Quartile 4 | 0.95 (0.89-1.01) | 0.97 (0.90-1.05) | 0.98 (0.91-1.06) | 1.01 (0.93-1.10) |
|  |  |  |  |  |
| HDI z score |  |  |  |  |
| Quartile 1 | Ref. | Ref. | Ref. | Ref. |
| Quartile 2 | 1.09 (1.03-1.17) | 1.10 (1.02-1.18) | 1.10 (1.03-1.18) | 1.10 (1.02-1.19) |
| Quartile 3 | 1.02 (0.95-1.09) | 1.02 (0.95-1.10) | 1.02 (0.95-1.10) | 1.03 (0.95-1.12) |
| Quartile 4 | 1.02 (0.95-1.09) | 1.04 (0.96-1.12) | 1.04 (0.97-1.13) | 1.06 (0.97-1.14) |

Abbreviations: Ref, reference group; BMI, body mass index; CVD, cardiovascular disease; CI, confidence interval; HDI, healthy diet indicator; HR, hazard ratio; MEDAS, Mediterranean Diet Adherence; MIND, Mediterranean-Dietary Approaches to Stop Hypertension Intervention for Neurodegenerative Delay; RFS, recommended food score; SBP, systolic blood pressure; WC, waist circumference.

Model 1: adjusted for age, sex, Townsend deprivation index, and ethnicity;

Model 2: additionally adjusted for smoking status, weekly units of alcohol use, physical activity, total sedentary time, sleep duration, total energy intake;

Model 3: additionally adjusted for number of long-term conditions.

Model 4: additionally adjusted for BMI, WC, total cholesterol, and SBP.

**Figure S1** **Directed Acyclic Graph of association between diet score and CVD and arrhythmias as shown in DAGitty.**

Each circle (node) represents a variable. The green node with an arrowhead inside is the exposure of interest, while the node filled in blue with an “I” inside is the main outcome of interest. Pink nodes correspond to the minimal set of confounders to adjust to remove biasing pathways. Blue nodes are potential mediators of the effect. Purple arrows show the direction of association between variables, while green arrows are potential causal paths between exposure and outcome.

**Figure S2 Dose-response association between standardized diet quality and incident main CVD subtypes**

The model was adjusted for age, sex, Townsend deprivation index, ethnicity, smoking status, weekly units of alcohol use, physical activity, total sedentary time, sleep duration, total energy intake, BMI, WC, total cholesterol, SBP, number of long-term conditions at baseline. BMI, body mass index; CI, confidence interval; HF, heart failure; HDI, healthy diet indicator; HR, hazard ratio; IHD, ischemic heart disease; SBP, systolic blood pressure; WC, waist circumference.

**Supplemental Figure 3 Dose-response association between standardized diet quality and incident cardiac arrhythmias subtypes**

The model was adjusted for age, sex, Townsend deprivation index, ethnicity, smoking status, weekly units of alcohol use, physical activity, total sedentary time, sleep duration, total energy intake, BMI, WC, total cholesterol, SBP, number of long-term conditions at baseline. BMI, body mass index; CI, confidence interval; HDI, healthy diet indicator; HR, hazard ratio; SBP, systolic blood pressure; WC, waist circumference
